# A Rare Mutation in *SMAD9* Associated With High Bone Mass Identifies the SMAD‐Dependent BMP Signaling Pathway as a Potential Anabolic Target for Osteoporosis

**DOI:** 10.1002/jbmr.3875

**Published:** 2019-11-14

**Authors:** Celia L Gregson, Dylan J. M. Bergen, Paul Leo, Richard B Sessions, Lawrie Wheeler, April Hartley, Scott Youlten, Peter I Croucher, Aideen M McInerney‐Leo, William Fraser, Jonathan CY Tang, Lisa Anderson, Mhairi Marshall, Leon Sergot, Lavinia Paternoster, George Davey Smith, The AOGC Consortium, Matthew A Brown, Chrissy Hammond, John P Kemp, Jon H Tobias, Emma L Duncan

**Affiliations:** ^1^ Musculoskeletal Research Unit, Translational Health Sciences Bristol Medical School, University of Bristol Bristol UK; ^2^ School of Physiology, Pharmacology, and Neuroscience, Faculty of Life Sciences University of Bristol Bristol UK; ^3^ Faculty of Health, Translational Genomics Group Institute of Health and Biomedical Innovation, Queensland University of Technology (QUT), Translational Research Institute, Princess Alexandra Hospital Woolloongabba Australia; ^4^ Faculty of Life Sciences School of Biochemistry, University of Bristol Bristol UK; ^5^ Medical Research Council Integrative Epidemiology Unit, Population Health Sciences Bristol Medical School, University of Bristol Bristol UK; ^6^ Division of Bone Biology Garvan Institute of Medical Research Sydney Australia; ^7^ Faculty of Medicine St Vincent's Clinical School, UNSW Sydney Sydney Australia; ^8^ School of Biotechnology and Biomolecular Sciences, UNSW Sydney Sydney Australia; ^9^ Dermatology Research Centre, The University of Queensland, The University of Queensland Diamantina Institute Brisbane Australia; ^10^ Norwich Medical School, University of East Anglia Norwich UK; ^11^ Department of Diabetes Endocrinology and Clinical Biochemistry, Norfolk and Norwich University Hospital NHS Foundation Trust Norwich UK; ^12^ Severn School of Radiology, Severn Deanery Bristol UK; ^13^ Faculty of Medicine The University of Queensland Diamantina Institute, The University of Queensland Woolloongabba Australia; ^14^ Department of Endocrinology and Diabetes Royal Brisbane & Women's Hospital Herston Australia; ^15^ Faculty of Medicine University of Queensland Herston Australia

**Keywords:** DXA, EXON SEQUENCING, HIGH BONE MASS, MONOGENIC, OSTEOANABOLIC, *SMAD9*, ZEBRAFISH

## Abstract

Novel anabolic drug targets are needed to treat osteoporosis. Having established a large national cohort with unexplained high bone mass (HBM), we aimed to identify a novel monogenic cause of HBM and provide insight into a regulatory pathway potentially amenable to therapeutic intervention. We investigated a pedigree with unexplained HBM in whom previous sequencing had excluded known causes of monogenic HBM. Whole exome sequencing identified a rare (minor allele frequency 0.0023), highly evolutionarily conserved missense mutation in *SMAD9* (c.65T>C, p.Leu22Pro) segregating with HBM in this autosomal dominant family. The same mutation was identified in another two unrelated individuals both with HBM. In silico protein modeling predicts the mutation severely disrupts the MH1 DNA‐binding domain of SMAD9. Affected individuals have bone mineral density (BMD) *Z*‐scores +3 to +5, mandible enlargement, a broad frame, torus palatinus/mandibularis, pes planus, increased shoe size, and a tendency to sink when swimming. Peripheral quantitative computed tomography (pQCT) measurement demonstrates increased trabecular volumetric BMD and increased cortical thickness conferring greater predicted bone strength; bone turnover markers are low/normal. Notably, fractures and nerve compression are not found. Both genome‐wide and gene‐based association testing involving estimated BMD measured at the heel in 362,924 white British subjects from the UK Biobank Study showed strong associations with *SMAD9* (P_GWAS_ = 6 × 10^−16^; P_GENE_ = 8 × 10^−17^). Furthermore, we found Smad9 to be highly expressed in both murine cortical bone–derived osteocytes and skeletal elements of zebrafish larvae. Our findings support *SMAD9* as a novel HBM gene and a potential novel osteoanabolic target for osteoporosis therapeutics. SMAD9 is thought to inhibit bone morphogenetic protein (BMP)‐dependent target gene transcription to reduce osteoblast activity. Thus, we hypothesize *SMAD9* c.65T>C is a loss‐of‐function mutation reducing BMP inhibition. Lowering *SMAD9* as a potential novel anabolic mechanism for osteoporosis therapeutics warrants further investigation. © 2019 The Authors. *Journal of Bone and Mineral Research* published by American Society for Bone and Mineral Research.

## Introduction

Age‐related bone loss with deterioration of skeletal architecture leads to osteoporosis, affecting 8.2 million women and 2.0 million men aged 50 years and older in the United States (US).^1^ Worldwide, osteoporosis causes more than 8.9 million fractures annually.^1^ Osteoporotic fractures and their treatment are a major cause of morbidity and mortality, with annual US health care costs exceeding $20 billion.^2^ Most osteoporosis treatment approaches, including all oral medications, reduce bone resorption and prevent further bone loss, rather than enhance bone formation. Affordable anabolic treatments, which can restore bone mass and skeletal architecture, are much needed.

Romosozumab, a monoclonal antibody against sclerostin, represents a new class of anti‐osteoporosis drug, recently approved by the FDA.^3^, ^4^ Sclerostin, a key inhibitor of bone formation, was discovered through study of two rare syndromes of extreme high bone mass (HBM) due to mutations in *SOST*.^5^, ^6^ *SOST* encodes Sclerostin, which binds to low‐density lipoprotein receptor‐related proteins 5 and 6 (LRP5 and LRP6) to prevent activation of canonical WNT signaling in bone, resulting in decreased bone formation. Gain‐of‐function mutations in *LRP5* and *LRP6* can also cause extreme HBM.^7^, ^8^ Together these sclerosing bone dysplasias are characterized by mandible enlargement with tori of the palate and mandible, bone overgrowth leading to nerve compression, a tendency to sink when swimming, and, importantly, resistance to fracture.^5^, ^7^, ^9^ These important gene discoveries validate the study of rare monogenic HBM as an approach to identify novel therapeutic targets for drug development toward osteoporosis treatments.

We have previously shown that HBM (defined as a total hip and/or first lumbar vertebral bone mineral density [BMD] *Z*‐score of ≥ +3.2) is observed in 0.18% of dual‐energy X‐ray absorptiometry (DXA) scans in the UK.^10^ Most cases are unexplained; ie, they do not carry mutations in established HBM genes.^9^ Although such HBM populations do show enrichment for common variant associations with established BMD‐associated loci,^11^ we hypothesized that novel causes of monogenic HBM remain to be determined. Thus, we aimed to identify novel monogenic causes of HBM to provide insight into regulatory pathways amenable to therapeutic intervention.

## Materials and Methods

### The UK HBM cohort

The HBM study is a UK‐based multicenter observational study of adults with unexplained HBM, identified incidentally on routine clinical DXA scanning. Full details of DXA database screening and participant recruitment have been reported^10^ (Supplemental Methods in File S1). In brief, DXA databases containing 335,115 DXA scans across 13 UK centers were searched; all scans explained by artefact or known causes of high BMD were excluded. Unexplained HBM was defined as 1) first lumbar vertebra (L_1_) Z‐score of ≥ +3.2 plus total hip (TH) *Z*‐score of ≥ +1.2 and/or 2) TH *Z*‐score ≥ +3.2 plus L_1_ *Z*‐score of ≥ +1.2. A total of 533 unexplained HBM cases were invited to participate; 248 (47%) were recruited. They passed on study invitations to first‐degree relatives and spouse/partner(s). Finally, 236 of 893 (26.4%) invited relatives were recruited, as were 61 of 217 (28.1%) invited spouses/partners.^10^ All participants underwent structured clinical assessment and DXA scanning (Supplemental Methods in File S1). Peripheral quantitative computed tomography (pQCT) scans were performed at the distal and mid‐shaft of the tibia (4% and 66% from distal endplate) in the nondominant lower limb using a Stratec XCT2000L (Stratec Medizintechnik, Pforzheim, Germany) as published previously^12^ (Supplemental Methods in File S1). Two non‐fasted EDTA samples were collected and serum separated and frozen within 4 hours to −80°C. Bone formation (Procollagen type 1 amino‐terminal propeptide [P1NP], total osteocalcin) and resorption (β‐C‐telopeptides of type I collagen [βCTX]) markers and sclerostin were measured (Supplemental Methods in File S1). DNA was extracted from peripheral venous blood using standard phenol/ chloroform extraction. Sanger sequencing of all HBM index cases for exons 2, 3, and 4 of *LRP5*, *SOST* (including the van Buchem disease deletion), and *LRP4* (exons 25 and 26) excluded seven individuals with *LRP5* mutations and one with a *SOST* mutation, leaving 240 unexplained HBM individuals.^9^

### Anglo‐Australasian Osteoporosis Genetics Consortium (AOGC) HBM and LBM cases

The original AOGC extreme truncate population included 1128 Australian, 74 New Zealand, and 753 British women, aged 55 to 85 years, ≥5 years postmenopausal, with either HBM (age‐ and sex‐adjusted TH BMD *Z*‐scores +1.5 to +4.0, *n* = 1055) or low bone mass (LBM) (*Z*‐scores −4.0 to −1.5, *n* = 900).^13^ LBM cases were excluded if they had secondary causes of osteoporosis (as previously described^13^). Unrelated samples of white ancestry with complete height and weight data and enough high‐quality genomic DNA were available in 947 individuals (426 AOGC high and 521 AOGC low BMD), from which (computation capacity limited sample size) the most extreme HBM cases were selected using a threshold TH or LS *Z*‐score ≥ +2.5, and the most extreme LBM cases using a LS *Z*‐score ≤ −0.5, so 126 HBM and 493 LBM samples were chosen to undergo whole exome sequencing (WES).

### Whole exome sequencing

Sequencing libraries for 859 samples (240 UK HBM, 126 AOGC HBM, and 493 AOGC LBM) were constructed. Base calling, sequence alignment and variant calling were performed as previously described^14^ (details in Supplemental Methods in File S1).

#### *Filtering pipeline applied to unexplained HBM pedigrees*

After quality‐filtering, data were analyzed for carriage of at least one rare (either novel or maximum population‐based minor allele frequency [MAF] <0.005) nonsynonymous single‐nucleotide variant (SNV) or indel in a highly conserved region (GERP score < 1.5) of a gene, carried by the affected individuals and not carried by unaffected individuals (ie, autosomal dominant carriage model). Data were then filtered based on functional prediction of SNVs using Polyphen^15^ to identify “probably damaging” and SIFT^16^ “deleterious” SNVs. Compound heterozygous and homozygous inheritance were also assessed.

### Sanger sequencing validation of pedigree‐based HBM mutation

Polymerase chain reaction (PCR) amplification of identified exons was performed on 50 ng genomic DNA (see Supplemental Methods 4 in File S1). Electropherograms were aligned and analysed using sequence analysis software Genalys (Version 2.0 ß, Masazumi Takahashi).

### Multi‐marker analysis of GenoMic annotation (MAGMA) in UK Biobank

Gene‐based tests of association were performed on 362,924 unrelated white British subjects (54% female) from the UK Biobank study with ultrasound‐derived heel estimated BMD (eBMD) and high‐quality genomewide HRC and 1000G/UK10K imputed data (Supplemental Methods in File S1). Detailed methodology has been published.^17^ Gene‐based tests of association were implemented in MAGMA v1.06^18^ using a multimodel approach combining association results from three separate gene analysis models: principal components regression, single‐nucleotide polymorphism (SNP)‐wise mean chi‐square model (ie, test statistic derived as sum of ‐log(SNP *p* value)) and SNP‐wise top chi‐square model (test statistic derived as sum of ‐log(SNP *p* value) for top SNPs) to produce an aggregate *p* value corresponding to the association between each of the 19,361 protein coding genes (±20 kb) and BMD, adjusting for age, sex, genotyping array, assessment center, and 20 ancestry informative principal components, with gene‐based significance threshold (*p* < 2.87 × 10^−6^).^17^

### Phenomewide association study (PheWAS)

PheWAS was conducted using GWASATLAS (https://atlas.ctglab.nl/), an online database of publicly available summary results statistics from 4,155 GWAS from 295 unique studies across 2960 unique traits and 27 domains.^19^ Significance for pleiotropic associations used a traditional genome‐wide significance threshold for SNP‐trait PheWAS (*p* < 5 × 10^−8^).^17^

### Gene expression in murine osteocytes

Whole transcriptome sequencing data from the primary osteocytes of four different bone types (tibia, femur, humerus, and calvaria) from mice (marrow removed, *n* = 8 per bone) were analyzed. A threshold of expression was determined based on the distribution of normalized gene expression for each sample.^20^ “Expressed” genes were those exceeding this threshold for all 8 of 8 replicates in any bone type. Osteocyte‐enriched expression of these genes in the skeleton was determined by comparing transcriptome‐sequencing data from bone samples with osteocytes isolated versus those samples with marrow left intact (*n* = 5 per group).^21^

### Replication in high BMD populations

WES data from AOGC were analyzed to identify any individual who carried the same rare (MAF < 0.025) mutation as identified from analysis of the HBM pedigree. Polyphen^15^ and SIFT,^16^ PMut^22^ and MutationTaster^23^ were used for in silico functional prediction. When the same point mutation was identified in more than one individual, haplotypes were compared between index case samples genotyped using an Infinium OmniExpress‐12v1.0 GWAS chip read using an Illumina iScan (San Diego, CA, USA), with genotype clustering performed using Illumina BeadStudio software.

### Protein structural modeling

The amino‐acid sequence of human SMAD9 was passed to the HHPred server.^24^ This located the best template structures in the Protein Databank for the MH1 domain, 5×6G (mouse SMAD5; 92% identity), and the MH2 domain, 3GMJ (*Drosophila melanogaster* MAD; 75% identity). Modeler was used to build the domain models according to the HHPred alignments.^25^ Chimera was used to introduce point mutations and remodel the domain swapping in the SMAD9‐MH1 model.^26^

### Zebrafish studies

*BMPre:GFP* (*Tg(5xBMPRE‐Xla*.*Id3:GFP)*)^27^ and *sp7:GFP* (*Tg(Ola*.*sp7:NLS‐GFP)*)^28^ transgenic fish (in London AB background) were housed and maintained in standard conditions.^29^, ^30^ Experiments were approved by the University of Bristol Animal Welfare and Ethical Review Body (AWERB) and performed in accordance with a UK Home Office project license. Developmentally staged larvae (after euthanization in MS222) were fixed in 4% paraformaldehyde (1 hour), dehydrated to 100% methanol, and stored at −20°C before staining. Immunolabeling was as previously described.^31^ Primary antibodies were anti‐Smad9 (rabbit polyclonal, Abcam, Cambridge, MA, USA, ab96698) used at a 1/100 dilution and anti‐GFP (chicken polyclonal, Abcam, ab13970) used at a 1/200 dilution in blocking buffer (5% horse serum). Secondary antibodies were used (A21206 and A11041, Invitrogen, Carlsbad, CA, USA) in a 1/400 dilution and samples incubated with DAPI (Sigma‐Aldrich, St. Louis, MO, USA, 1/1000 dilution) to visualize nuclei. Samples were mounted in 1% low melting point agarose and imaged with a confocal laser scanning microscope (Leica, Buffalo Grove, IL, USA, SP5II AOBS attached to a Leica DM I6000 inverted epifluorescence microscope) using a 40× PL APO CS (1.3 numerical aperture) lens. Images were processed and color balanced in Fiji.^32^

## Results

### HBM pedigree with a segregating *SMAD9* c.65T>C p.Leu22Pro variant

**Figure 1:**
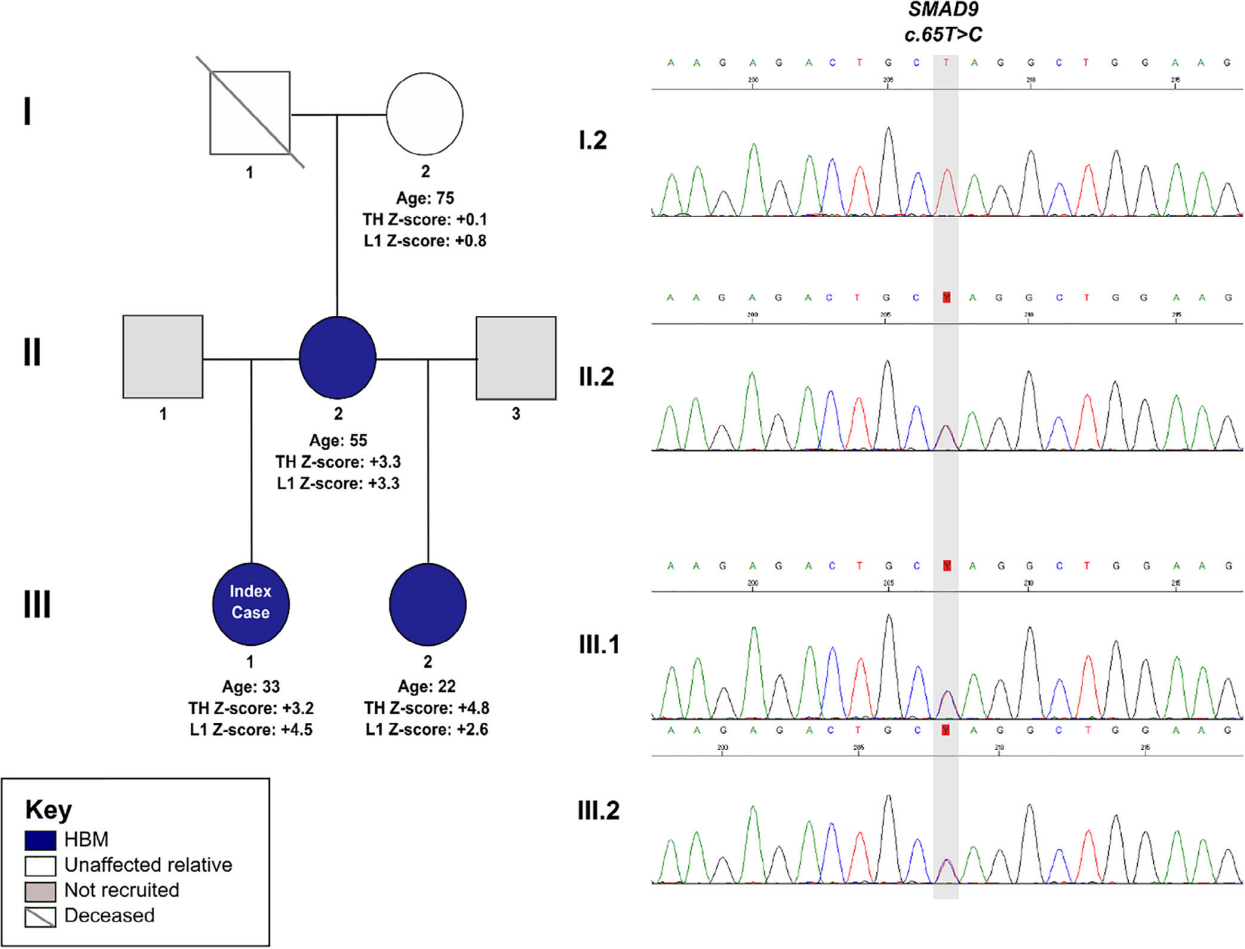
The HBM pedigree and electrophoretogram images of a segregating *SMAD9* c.65T>C, p.Leu22Pro variant.

We investigated a pedigree with unexplained and apparently autosomal dominant HBM (Fig. 1),^9^ identified from our large UK HBM cohort^10^ (Fig. S2).

#### *Clinical phenotype*

Clinical phenotypes of the UK family individuals are shown in Table 1; extended clinical histories are provided in Supplemental Results. In summary, affected individuals had high BMD *Z*‐scores and very high body mass index (BMI) and had not had any adult fractures, nerve compression, or dental problems; however, bone pain was common, without a clear cause. There was no clinical history of intellectual impairment, pulmonary hypertension, vascular hypertension, hematological abnormalities, pubertal delay, or other clinical conditions. None had been exposed to anabolic or antiresorptive medications.

**Table 1:** Characteristics of the c.65T>C, p.Leu22Pro SMAD9 High Bone Mass (HBM) Pedigree Members and Two Further Unrelated HBM Individuals With the Same SMAD9 Mutation

	HBM pedigree				Additional isolated HBM cases		
	UK proband III.1	UK half‐sister III.2	UK mother II.2	UK grandmother I.2 (unaffected)	UK case	Australian case
*SMAD9* mutation	Leu22Pro	Leu22Pro	Leu22Pro	WT	Leu22Pro	Leu22Pro
Age (years) at assessment	33	22	55	75	55	57
Sex	Female	Female	Female	Female	Female	Female
Ethnicity	White	White	White	White	White	White
Anthropometry						
Height (cm)	178.0	173.3	175.0	160.6	160.0	162.2
Weight (kg)	138.0	133.8	127.8	72.6	89.7	69.9
Body mass index (kg/m^2^)	43.6	43.3	41.4	28.1	35.0	26.6
DXA measurements						
Total hip BMD *Z*‐score	+3.2	+4.8	+3.3	+0.1	+4.3	+2.7
L_1_ BMD *Z*‐score	+4.5	+2.6	+3.3	+0.8	+5.0	+3.4*
Bone mineral content (kg)	3.49	3.77	3.65	2.12	3.24	‐
Fat mass (kg)	73.2	64.5	64.8	25.4	34.0	‐
Lean mass (kg)	61.3	65.6	59.5	45	52.4	‐
Clinical phenotype						
Adult fracture	No	No	No	No	No	No
Sinks/floats	Floats	Sinks	Floats	Floats	Sinks	‐
Bone pain	Yes	Yes	Yes	No	No	‐
Visual/auditory impairment	Myopia	No	No	Impaired hearing	Astigmatism	‐
Dentition	Normal	Normal	Normal	Normal	Retained cuspid tooth	‐
Shoe size^a^	10	10	9	6	5.5	‐
Broad frame	Yes	Yes	Yes	No	Yes	‐
Enlarged mandible	Yes	Yes	Yes	No	Yes	‐
Torus	Yes	Yes	No	No	No	‐
Nerve compression	No	No	No	No	No	‐
Pes planus	No	No	Yes	No	Yes	‐
Blood tests						
ALP (IU/L)	99	83	102	202	61	‐
Adjusted calcium (mmol/L)	2.50	2.46	2.40	2.46	2.33	‐
P1NP (ug/L)	58	36	22	95	35	‐
CTX (ug/L)	0.15	0.18	0.19	0.10	0.16	‐
Osteocalcin (ug/L)	12.4	17.1	13.5	14.8	11.5	‐
Sclerostin (pmol/L)	71.0	‐	56.1	44.4	50.4	‐

WT = wild type; DXA = dual‐energy X‐ray absorptiometry; BMD = bone mineral density.

a UK measurements. Reference ranges: ALP 20–120; adjusted calcium 2.25–2.70; P1NP: premenopausal 30–78 ug/L, postmenopausal 26–110 ug/L, male 20–76 ug/L; serum CTX 0.1–0.5 ug/L; osteocalcin 6.8–32.2 ug/L; sclerostin <80 pmol/L.

* measured from L1‐4.

#### *III.1: Index case (c.65T>C, p.Leu22Pro)*

The 33‐year‐old index case, with BMD *Z*‐scores +3.2 at the total hip and +4.5 at first lumbar vertebra (L_1_), had only sustained one traumatic fracture at age 20 months. She reported lower leg and ankle pain. She was tall (>97th centile) and obese, with increased shoe size, a broad frame, enlarged mandible, and a 4 mm torus mandibularis. She had normal joints. Radiographs showed increased cortical thickness and new bone formation at the anterior inferior iliac spines bilaterally (Supplemental Fig. S1 in File S1).

#### *II.2: Mother of the index case (c.65T>C, p.Leu22Pro)*

The 55‐year‐old affected mother, with BMD *Z*‐scores +3.3 at the total hip and at L_1_, had never sustained a fracture. Six years earlier she had had a right calcaneal spur surgically removed. She had widespread pain with a diagnosis of fibromyalgia. She was tall (97th centile) and obese, with above average shoe size, a broad frame, enlarged mandible, but no tori. She had a full range of movement in all joints, bilateral knee crepitus, and bilateral pes planus.

#### *III.2: Half‐sister to index case (c.65T>C, p.Leu22Pro)*

The 22‐year‐old affected half‐sister, with BMD *Z*‐scores +4.8 at the total hip and +2.6 at L_1_, had not fractured. She had had sciatica for 5 years, lumbar back pain and fronto‐temporal headaches for 11 years, with a diagnosis of migraine. She was tall (93rd centile) and obese, with above average shoe size, a broad frame, enlarged mandible, a torus palatinus in the midline of her hard palate (3 cm × 7 mm), and normal joint movement.

#### *I.2: Grandmother of index case (wild type)*

The 75‐year‐old grandmother, who did not have HBM, had also never sustained a fracture. She had widespread osteoarthritis and on examination had reduced extension of the right elbow and left knee, and bilateral knee crepitus. However, in contrast to other family members, she was less overweight with normal shoe size, frame, mandible, and no tori.

#### *Sequencing of pedigree*

WES identified a heterozygous missense variant in *SMAD9* (SMAD family Member 9 referring to homologies to the Caenorhabditis elegans SMA (small worm phenotype) and Drosophila MAD (“Mothers Against Decapentaplegic”)) (NM 001127217: exon2: c.65T>C, p.Leu22Pro), segregating with HBM (ie, present in all three individuals with HBM (*III*.*1*, *II*.*2*, *III*.*2*) but absent from *I*.*2* (Fig. S2). This variant (rs111748421) is rare (Exome Aggregation Consortium [ExAC] minor allele frequency [MAF] 0.0023 in European non‐Finnish populations), affects a highly evolutionarily conserved base (genomic evolutionary rate profiling [GERP] 5.53), and is predicted to be pathogenic by multiple protein‐prediction algorithms (deleterious by SIFT,^16^ probably damaging by Polyphen,^15^ and disease causing by MutationTaster^23^ and PMut^22^).

A novel variant in *CHRNA1* (cholinergic receptor, nicotinic, alpha 1) (c.560T>C, p.Leu187Pro) was also identified (GERP 5.29). Mutations in *CHRNA1* have been associated with congenital myasthenic syndromes (OMIM#100690), not present in this pedigree. No variants were identified when applying a compound heterozygous or an autosomal recessive inheritance model.

### Sequencing of other HBM cases identifies two further isolated HBM cases harboring a c.65T>C, p.Leu22Pro variant

WES of a further 366 HBM cases (240 isolated cases from the UK cohort with a total hip (TH) or L_1_ *Z*‐score ≥+3.2 and 126 individuals from the Anglo‐Australasian Osteoporosis Genetics Consortium (AOGC)^33^ with either a total hip and/or lumbar spine (LS) *Z*‐score between +2.5 and +4.0) (Supplemental Fig. S2 in File S1) identified two individuals with the same *SMAD9* c.65T>C, p.Leu22Pro variant. Haplotypic analysis confirmed these women were neither related to each other nor to the pedigree described above.

#### *Clinical phenotype*

***Isolated HBM case (c***.***65T>C***, ***p***.***Leu22Pro) from the UK*** (Table 1; Supplemental Fig. S3 and Results in File S1). This 55‐year‐old female, with BMD *Z*‐scores +5.0 at the total hip and +4.7 at L_1_, had never fractured and had no symptoms of nerve compression. Her adult left upper cuspid tooth had never erupted; wisdom teeth had been extracted for overcrowding. She had noticed her own mandible enlargement. She had a congenital astigmatism of her left eye with poor vision and congenital bilateral pes planus. Height was normal (30th centile). She was obese with a broad frame, mandible enlargement, but no tori. She had normal joints.

#### *Isolated HBM case (c.65T>C, p.Leu22Pro) from Australia*

This 57‐year‐old female, with BMD *Z*‐scores +3.0. at the total hip and +2.7 at L_1_, reported a nose fracture as a child. Height was on the 45th centile and she was overweight. She did not have any history of conditions affecting bone health and had not received antiresorptive or anabolic medications. No further clinical details were available.

### Tibial pQCT evaluation

All members of the HBM pedigree, plus the additional isolated HBM case from the UK underwent pQCT scanning of the tibia (Table 2; Supplemental Table S1 and Supplemental Fig. S4 in File S1). To set these findings in context, the mean (SD) values from the four c.65T>C, p.Leu22Pro *SMAD9* HBM cases were compared against values from 76 unrelated female HBM cases (without *SMAD9*, *LRP5*, *LRP4*, or *SOST* mutations) and 32 female family controls with normal DXA‐measured BMD who had had pQCT scans following the same protocol.^12^ The four *SMAD9* HBM cases had greater trabecular density, cortical area and thickness, and predicted bone strength (strength stain index [SSI]) than other HBM cases and, to a greater extent, than unaffected family controls. Muscle size (cross‐sectional area) was also notably larger in the *SMAD9* HBM group (Table 2).

**Table 2:** Distal and Mid‐Shaft Tibial pQCT Measures in High Bone Mass (HBM) Cases Compared With Female HBM Cases Without *SMAD9, LRP5, LRP4, SOST* Mutations, and Female Family Controls With Normal BMD

		*SMAD9* HBM cases Leu22Pro *n* = 4 Mean (SD)	WT female HBM cases^a^ *n* = 76 Mean (SD)	*p* Value^b^	Female family controls with normal BMD *n* = 32 Mean (SD)	*p* Value^c^
	Age (years)	41.3 (16.5)	60.8 (12.3)		54.8 (13.5)	
	Total hip BMD *Z*‐score	+3.8	+2.9		+0.39	
4% distal tibia	Total bone area (mm^2^)	1038 (160.6)	1052 (122.6)	0.820	817.1 (223.5)	0.066
	Trabecular BMD (mg/cm^3^)	342.3 (13.3)	324.3 (22.5)	0.118	308.0 (24.6)	0.010
	Cortical thickness (mm)	2.12 (0.79)	1.04 (0.81)	0.011	0.87 (0.83)	0.007
66% mid‐shaft tibia^d^	Total bone area (mm^2^)	608.3 (4.7)	601.5 (81.9)	0.886	572.7 (73.9)	0.416
	Cortical BMD (mg/cm^3^)	1150 (10.1)	1126 (35.9)	0.255	1111 (65.7)	0.319
	Cortical thickness (mm)	4.96 (0.13)	4.37 (0.62)	0.104	3.80 (0.71)	0.008
	Cortical bone area (mm^2^)	356.3 (9.1)	316.6 (36.6)	0.065	274.1 (42.4)	0.002
	Cortical/total bone area (%)	58.6 (1.1)	53.3 (7.57)	0.236	48.3 (8.34)	0.043
	SSI (mm^3^)	1680 (21.1)	1506 (236.6)	0.211	1298 (248.2)	0.013
	Muscle area (mm^2^)	8334 (536.5)	6939 (980.8)	0.017	6542 (1033)	0.006
	Muscle density (mg/cm^3^)	42.1 (1.5)	40.1 (4.0)	0.392	40.2 (3.1)	0.323

BMD = bone mineral density; SD = standard deviation; SSI = strength strain index; WT = wild type.

a Female subgroup (without *SMAD9*, *LRP5*, *LRP4*, *SOST* mutations) analyzed using data previously published, collected, and analyzed with the same protocols as *SMAD9* HBM cases.^9^

b Analysis of *SMAD9* HBM cases versus WT HBM cases.

c Family controls with normal BMD.

d *n* = 3.

### Sequencing of low bone mass (LBM) cases

WES data from 473 women with LBM from the AOGC consortium with TH *Z*‐scores between −1.5 and −4.0 and a LS *Z*‐score ≤−0.5, obtained using similar methodology to the AOGC HBM cases, was interrogated (Supplemental Fig. S2 in File S1). The c.65T>C, p.Leu22Pro *SMAD9* variant was not observed.

### Common *SMAD9*‐associated genetic variants and BMD

**Figure 2:**
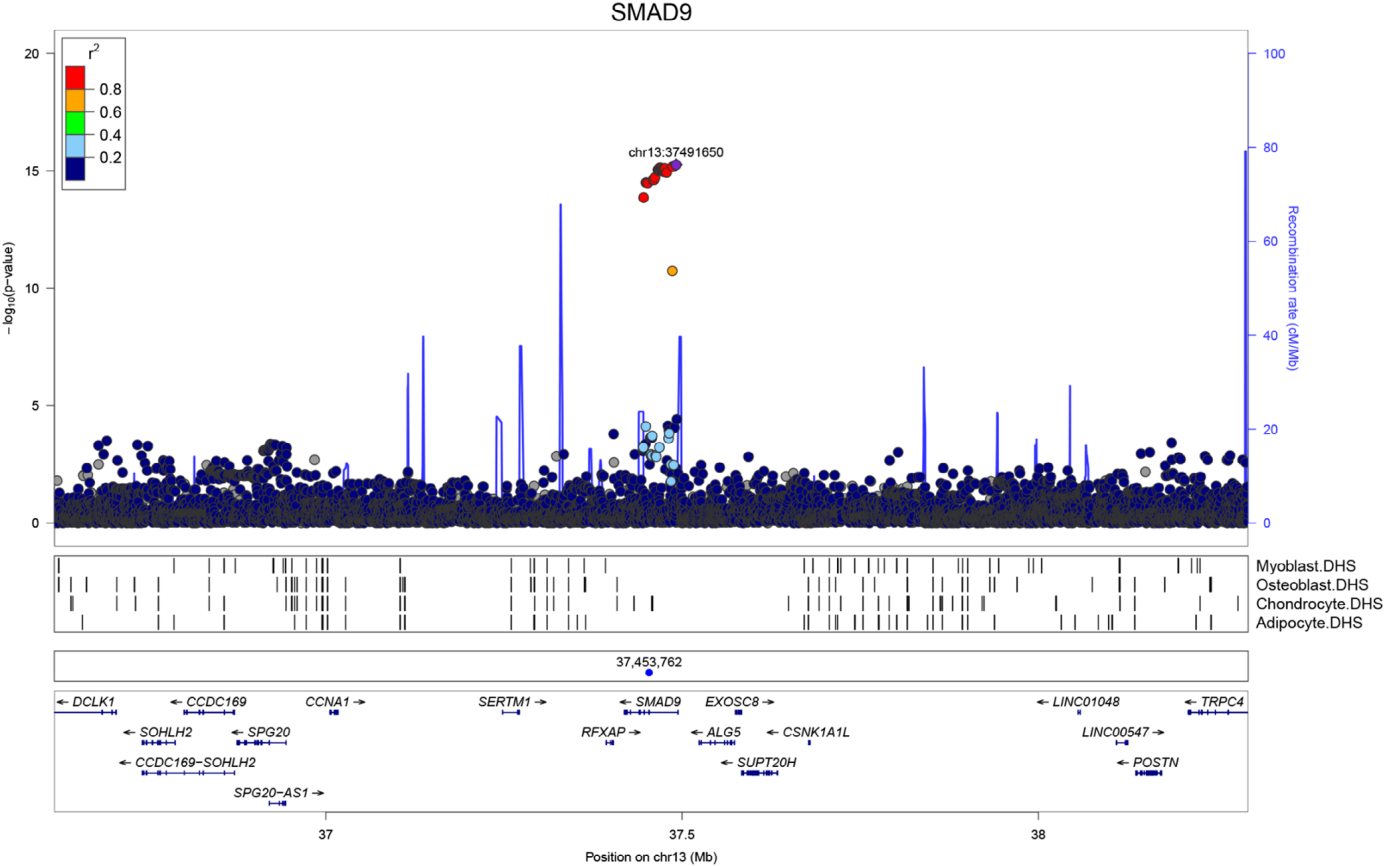
GWAS for eBMD measured in UK Biobank: Regional association plot for the locus containing *SMAD9*. Top panel: circles show unconditioned GWAS *p* values and genomic locations of imputed SNPs within ±800 kb of the 5′ and 3′ UTR of each gene. Colors indicate varying degrees of pairwise linkage disequilibrium between the lead eBMD‐associated SNP (rs12427846, purple diamond) and all other SNPs. Second panel: Vertical shaded areas correspond to locations of DNase I hypersensitive sites (DHSs) characteristic of skeletal muscle myoblasts cell line (E120), osteoblast primary cells (E129), mesenchymal stem cell–derived chondrocyte cultured cells (E049) and mesenchymal stem cell–derived adipocyte cultured cells (E023). Red shading depicts intersections between DHSs and genomewide significant SNPs. Black shading denotes instances in which any other SNPs intersect DHSs. Third panel: Blue circle shows the position of the putative causal variant c.65T>C, p.Leu22Pro. Fourth panel: Horizontal lines represent genes with vertical lines annotating location of exons. Arrows indicate the direction in which each gene is transcribed.

Publicly available data from a recent population‐based genomewide association study (GWAS) of eBMD (estimated BMD by heel ultrasound in the UK Biobank study)^17^ were used to investigate variants surrounding both *SMAD9* and *CHRNA1*. Regional association plots suggested that SNPs intersecting *SMAD9* are strongly associated with eBMD (lead SNP rs12427846 [MAF 0.25], β 0.02, SE 0.002, *p* = 5.5 × 10^−16^; Fig. 2). In contrast, SNPs surrounding *CHRNA1* were not robustly associated with eBMD (Supplemental Fig. S5 in File S1). These observations were further supported by gene‐based tests of association performed in‐house using 362,924 unrelated white British subjects from the UK Biobank Study. Specifically, *SMAD9* was more strongly associated with eBMD (P_JOINT_ = 7.94 × 10^−17^), when compared with neighboring genes within ±800 kb (*p* > 2.4 × 10^−2^) (Supplemental Table S2 in File S1). No such enrichment was found for *CHRNA1* (Supplemental Table S3 in File S1). Further investigation of rs12427846 in the UK Biobank Study identified weak associations with body weight (β −0.14, SE 0.04, *p* = 1.6 × 10^−3^) and with height (β −0.07, SE 0.03, *p* = 3.5 × 10^−3^) with effects in the opposite direction from that found with eBMD; however, adjustment for weight and height did not attenuate the strong association between rs12427846 and eBMD reported above. Interrogating the GWAS Catalog (https://www.ebi.ac.uk/gwas/) did not identify associations of the rare (MAF 0.0014) variant rs111748421 with any trait (neither bone‐related nor any other).

### Phenomewide association study

PheWAS involving nearly 3000 traits^19^ identified no clear evidence for pleiotropy for the c.65T>C, p.Leu22Pro *SMAD9* variant (rs111748421) (Supplemental Fig. S6 and Supplemental Table S4 in File S1). Analysis involving the common *SMAD9* variant (rs12427846) revealed robust pleiotropic associations with BMD traits. Similarly, a gene‐based PheWAS of *SMAD9* identified robust evidence of gene‐level pleiotropy with BMD. To investigate further possible pleiotropic associations with metabolic phenotypes, we looked up rs111748421 and rs12427846 in the Myocardial Infarction Genetics and CARDIoGRAM Exome meta‐analysis^34^, ^35^ and the subsequent meta‐analysis to which results from UK Biobank SOFT CAD GWAS and CARDIoGRAMplusC4D 1000 Genomes‐based GWAS were added.^36^ No association was found for rs111748421 in either. Although rs12427846 was not present in the first meta‐analysis, the eBMD‐increasing allele was only nominally associated with the composite cardiovascular disease outcome in the second (log OR 0.03 [SE 0.01], *p* = 9 × 10^−4^; significance threshold *p* < 5 × 10^−8^).^36^

### Smad9 expression in murine osteocytes

We next determined whether *Smad9* and *Chrna1* are expressed in osteocytes, the master cell regulators in the skeleton and key regulators of bone mass,^37^ and enriched in osteocytes compared with other cells in bone.^21^ Smad9 mRNA was highly expressed in murine osteocytes, whereas Chrna1 was not (Supplemental Table S5 in File S1).

### Smad9 expression in zebrafish skeletal tissue

**Figure 3:**
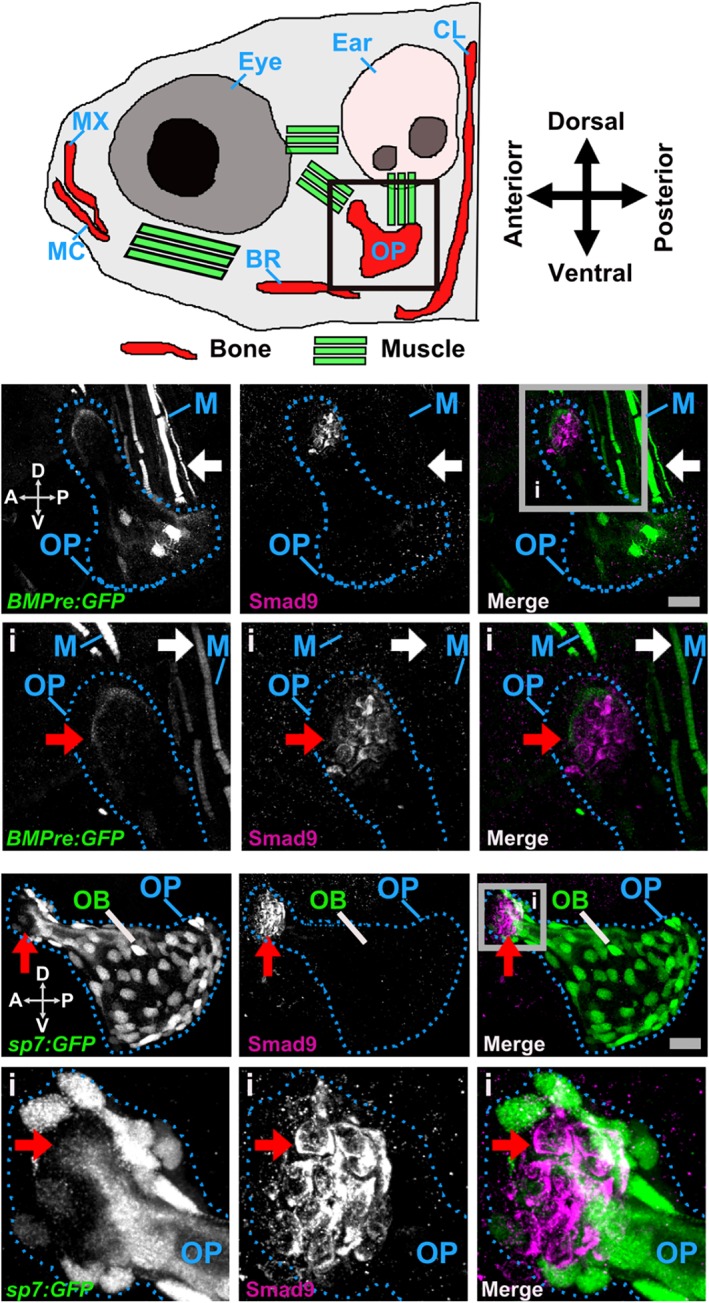
Smad9 protein expression in the larval zebrafish opercle bone. (*A*) Schematic of the larval zebrafish head (6 days post fertilization [dpf], lateral view), showing visible ossified elements (red) and the main muscle groups (green) that are green fluorescent protein (GFP)‐positive under control of the BMP‐responsive elements promoter (BMPre) transgenic reporter line (*BMPre:GFP*). The black box indicates the location of the intramembranous opercle bone as shown in *B* and *C*. (*B*) Distinct tissue distribution of Smad9‐ and BMP‐expressing cells (7 dpf). Upper left panel: *BMPre:GFP*‐positive cells (white) in the levator operculi muscle group (white arrow) and ventral (V) side of the opercle (OP; dotted blue outline); upper middle: distinct group of Smad9‐positive cells (white) in the dorsal (D) tip of the opercle; upper right: merged view showing distinct tissue expression of *BMPre:GFP*‐positive cells (green) and Smad9‐positive cells (purple); lower left: gray box inset (i) showing faint cap of *BMPre:GFP*‐positive cells at the dorsal tip of the opercle (red arrow); lower middle: cluster of Smad9‐positive cells; lower right: merged view confirming non‐overlapping distribution of *BMPre:GFP*‐positive cells and Smad9‐positive cells. Images from *n* = 4 larvae. (*C*) Distinct tissue distribution of Smad9‐ and osterix (Sp7)‐expressing cells (6 dpf). Upper left: *Sp7:GFP*‐positive osteoblasts (OB; white) within the opercle; upper middle: Smad9‐positive cells (white) in the antero (A)‐dorsal tip of the opercle (red arrow); upper right: merged view showing separation of *Sp7:GFP*‐positive cells (green) and Smad9‐positive cells (purple) (Supplemental Movie S1 in File S1); lower left: the inset (i, gray box) shows few *Sp7:GFP*‐positive osteoblasts within the dorsal tip of the opercle; lower middle: cluster of Smad9‐positive cells; lower right: merged view confirming non‐overlapping distribution of *Sp7:GFP*‐ and Smad9‐positive cells. Images from *n* = 6 larvae. (*B*, *C*) Scale bar = 20 μm; all are maximum‐intensity z‐projection confocal images. A = anterior; BR = branchiostegal ray; CL = cleithrum; D = dorsal; M = muscle; MC = Meckel's cartilage; MX = maxilla; OB = osteoblast; OP = operculum; P = posterior; V = ventral.

**Figure 4:**
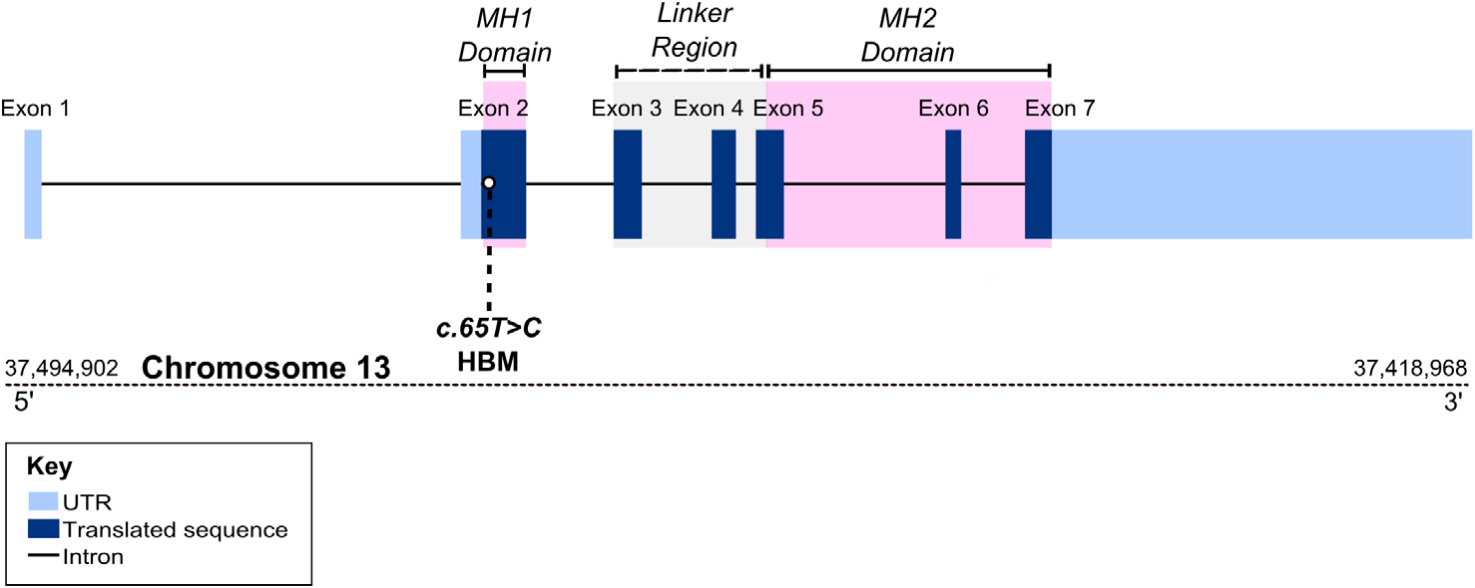
Position of c.65T>C p.Leu22Pro variant within *SMAD9*.

We also examined Smad9 protein expression in the developing zebrafish skeleton^38^ at 6 and 7 days post fertilization (dpf) (Fig. 3 *A*). A focus of Smad9 expression was observed at the dorsal tip of the opercle, an intramembranous bone overlying the gills, adjacent to but distinct from a region of bone morphogenetic protein (BMP) reporter activity (Fig. 3 *B*). The opercula muscle group also showed evidence of BMP reporter activity, whereas Smad9 expression at this site was absent. Smad9‐expressing cells in the opercle were negative for the osteoblast marker, sp7 (osterix), suggesting they are likely to represent pre‐osteoblasts (Fig. 4 *C* and Supplemental Movie S1 in File S1). Equivalent findings were observed in the branchiostegal ray bones and in the notochord at 6 and 7 dpf (Supplemental Fig. S7 in File S1).

### SMAD9 protein structural modeling

**Figure 5:**
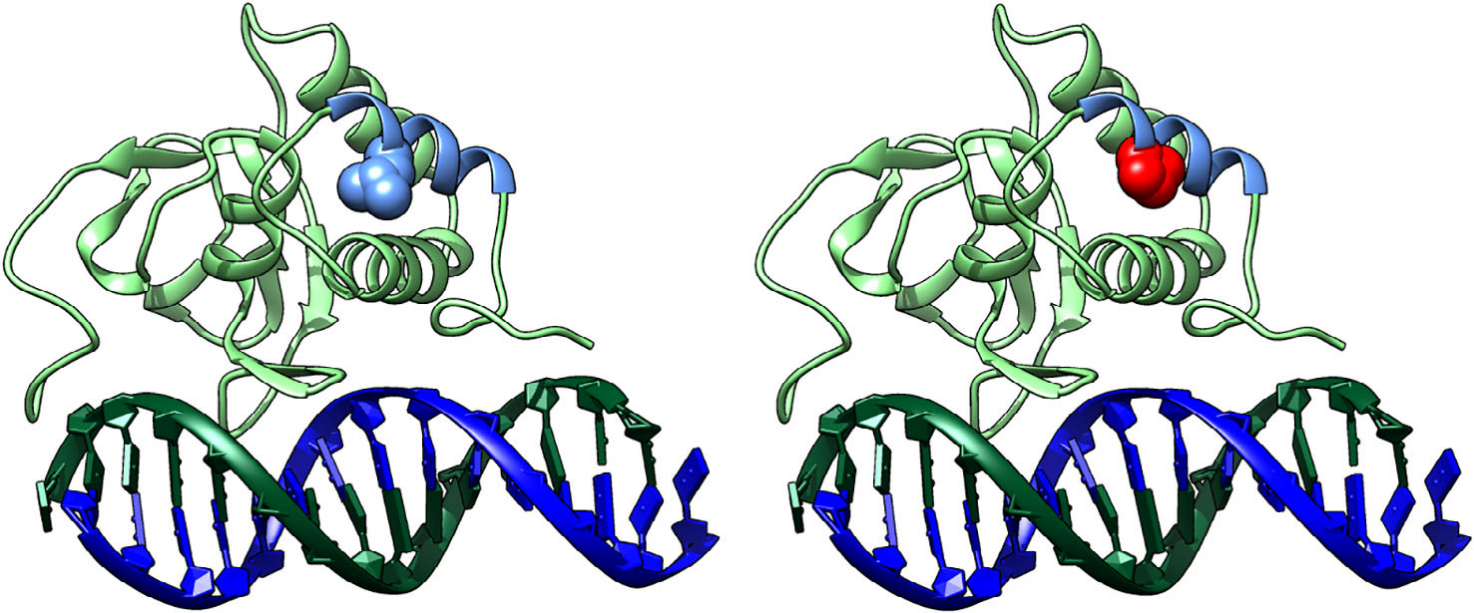
(*A*) Wild‐type (WT) *SMAD9* MH1 domain (light green ribbon with helix‐1 in light blue) binding the DNA helix (dark blue/dark green). L22 is shown in blue space filling. (*B*) L22P, shown in red space filling, is predicted to severely disrupt the structure of the MH1 domain. Supplemental Movie S2 in File S1: 3‐dimensional rotating image.

SMAD9 is a TGF‐beta family member DNA binding transcription factor. Phosphorylation by BMP‐ligand‐bound type 1 receptor kinase activates SMAD9, which translocates from the cytoplasm to the nucleus to regulate target gene expression.^39^ The seven exons of human *SMAD9* encode a protein of 467 amino acids that contains two MAD‐homology (MH) domains (MAD: Mother against Dpp) separated by a linker region (Fig. 4). The p.Leu22Pro *SMAD9* mutation is located within the MH1 domain responsible for DNA binding (Fig. 4) and lies in the hydrophobic face of the N‐terminal alpha helix (helix‐1) (Fig. 5, Supplemental Movie S2 in File S1). Helix‐1 packs against a groove made by helix‐2 and ‐3 within MH1, forming part of the hydrophobic core of this domain. Substitution of leucine by proline will: 1) introduce a less hydrophobic residue into this position; and 2) compromise the α‐helical fold by disrupting the canonical hydrogen bonding of helix‐1. Thus, modeling suggests that this mutation will disrupt the MH1 domain so severely that SMAD9 can no longer bind DNA and/or will be unstable, leading to protein degradation.

## Discussion

We report the first HBM pedigree with a segregating *SMAD9* mutation, with replication in two further unrelated individuals with HBM. *SMAD9* (also known as *SMAD8*, *MADH6*, and *MADH9*) encodes a downstream modulator of the BMP signaling pathway. BMPs, members of the TGF‐β superfamily, induce the formation of bone and cartilage.^40^ SMADs, activated by ligand‐binding of cell surface BMP receptors, mediate downstream intracellular signaling and biological responses induced by BMPs.^41^ Smad6 and Smad7 both inhibit BMP receptor activation and downstream signaling, as does Smad9 by more direct transcriptional repression.^39^ Our in silico protein modeling predicts that the p.Leu22Pro mutation severely disrupts the structure of the MH1 DNA binding domain of *SMAD9*, leading to loss of function.

Few previous studies have examined sites of Smad9 tissue expression. We have confirmed that Smad9 is expressed in mouse cortical bone‐derived osteocytes and the Smad9 protein in skeletal elements of zebrafish larvae. Moreover, we observed that BMP reporter activity in zebrafish was absent at sites of Smad9 expression, consistent with a functional role in BMP repression.^39^ Mutant mouse models with a *LacZ* insertion causing Smad9 truncation have not undergone BMD phenotyping; however, they have shown strong *LacZ* expression (under control of an endogenous *Smad9* promoter) within developing skeletal sites (eg, ribs, maxilla, mandible), gut, and lungs.^42^, ^43^ Taken together, our findings suggest that *SMAD9* c.65T>C is a loss‐of‐function mutation, causing HBM through a novel mechanism of enhanced bone formation due to reduced BMP inhibition.

Further, we have shown that the region containing *SMAD9* is strongly associated with BMD within the general population. Common variants intersecting *SMAD9* associate with population‐based measures of eBMD, as evidenced recently by fine‐mapping of target genes^17^, ^44^ and from our gene‐based tests of association presented here. Furthermore, rs12427846 (the lead SNP from these eBMD results) is associated with DXA‐measured total body BMD^45^ and fracture risk,^17^ also consistent with associations with BMD identified in our PheWAS. These findings provide further evidence of the importance of *SMAD9* in bone biology and are equivalent to reported associations for common variants annotated to *LRP5* and *SOST* genes, both similarly implicated in monogenic HBM disorders.^46^, ^47^

We have previously estimated unexplained HBM to have a prevalence of 0.181% amongst a DXA‐scanned adult population in the UK.^10^ As two of 248 cases fulfilling our stringent HBM phenotype definition (Supplemental Fig. S2 in File S1) were found to harbor the c.65T>C p.Leu22Pro *SMAD9* variant (rs111748421), we would estimate prevalence of *SMAD9* HBM as approximately 1 in 100,000 (1.46 × 10^−5^), less common than *LRP5* HBM.^9^ As rs111748421 has a reported MAF of 0.0028, this raises the possibility of incomplete penetrance, variable expressivity, gene–gene or gene–environment interaction with a currently unknown factor; it is also possible that rs111748421 might be in linkage disequilibrium (LD) with an intronic regulatory variant not captured by WES.

The clinical phenotype of c.65T>C, p.Leu22Pro *SMAD9* HBM includes mandible enlargement, a broad frame, torus palatinus, pes planus, increased shoe size, and, in 2 of 5 subjects, a tendency to sink when swimming. Adult fractures were not reported, raising the possibility of increased skeletal strength, supported by evidence of greater cortical bone and an increased strength‐strain index (SSI) quantified by pQCT (discussed further below), both of which promote fracture resistance. Mandible enlargement, torus palatinus, a tendency to sink when swimming, and an absence of adult fractures are reminiscent of *LRP5* HBM.^7^, ^48^ Encouragingly, unlike sclerosteosis (due to anabolic *SOST* mutations) and some *LRP5* HBM cases,^9^, ^49^ nerve compression was not a feature of *SMAD9* HBM.

The musculoskeletal phenotype of p.Leu22Pro *SMAD9* HBM includes high BMD *Z*‐scores (+3 to +5), with increased fat and lean mass. Although morbid obesity with increased stature was found in 3 of 5 *SMAD9* HBM cases, this was not ubiquitous to all. The increases in BMD of +3.3 to 5 SDs above normal exceeded increases in fat‐mass index of +0.9 to 3.4, supporting HBM as the primary phenotype, rather than high BMD occurring as a consequence of high fat mass.^50^ Furthermore, fat‐associated phenotypes were not identified in our PheWAS. pQCT revealed increased volumetric trabecular bone density with greater cortical thickness and area, suggesting reduced bone remodeling to reduce endosteal expansion. In support, bone turnover markers are at the lower end of the normal range. This phenotype mimics that previously described for human *LRP5* HBM^51^ although plasma sclerostin is not elevated, in contrast to *LRP5* HBM,^52^ suggesting that a negative feedback loop downregulating WNT signaling is not present. pQCT further identified larger muscle size in *SMAD9* HBM cases, including in the independent UK case with normal stature. This contrasts with findings from our zebrafish studies that Smad9 expression is absent from skeletal muscle tissue, as is also observed in murine models.^43^ Given the well‐recognized cross‐talk between muscle and bone^53^ and the large BMI of these individuals, it is conceivable that the increase in muscle size is secondary to a need to carry the substantial weight of both fat and bone mass. However, similar increases in muscle size have not been reported in other monogenic HBM conditions (ie, *LRP5* or *SOST* HBM) with equivalent BMD.

A clinical report of 13q13.3‐q21.3 deletion, leading to haploinsufficiency of *SMAD9* amongst other genes, identified a phenotype of skeletal overgrowth with infant height >95th percentile, consistent with the adult phenotype we describe, implicating *SMAD9* in the regulation of linear growth.^54^ We found limited, but consistent, evidence that *SMAD9* HBM may affect longitudinal growth. Although differences in height can artefactually affect DXA‐measured BMD, pQCT measures of increased trabecular bone density and cortical thickness are usually more independent of body size. Heterozygous truncating *SMAD9* mutations are associated with primary pulmonary hypertension (OMIM#615342),^55^ a phenotype not apparent in our HBM cases. Reported mutations affect a different domain from the mutation observed here, with p.Cys202X^55^ and p.Arg294X^56^ truncating the SMAD9 protein in the linker region between MH1 and MH2. A truncating mutation (p.Arg247X) has been associated with cerebral arteriovenous malformations in childhood.^57^ An activating heterozygous p.Val90Met germline mutation, affecting the 4th α‐helix of MH1 and close to the DNA binding interface, has been described in one pedigree with hamartomatous polyposis.^58^ In contrast to p.Leu22Pro, p.Val90Met appears to be a gain‐of‐function mutation, thought to arise from a steric clash, prompting a His104 residue to enhance DNA binding.^58^ Such examples of diverse phenotypes arising from mutations in differing exons of the same gene are well recognized, eg, differing mutations in *FBN1* (*Fibrillin 1*) can cause Marfan syndrome (with associated tall stature) (OMIM#154700), acromicric dysplasias (with short stature) (OMIM#102370), or stiff skin syndrome (OMIM#184900).^59^, ^60^, ^61^

We are only aware of one other skeletal dysplasia reported in association with an inhibitory SMAD (which include *SMAD6* and *SMAD7*). A rare *SMAD6* mutation has been associated with susceptibility to nonsyndromic midline craniosynostosis 7 (OMIM#617439), but only in the context of co‐inheritance of a common variant in *BMP2* strongly associated with this condition, a rare example of two locus inheritance.^62^ Interestingly, amongst the 1103 conditionally independent SNPs reaching genomewide significance in the UK Biobank eBMD GWAS (population *n* = 426,824), as well as identifying the *SMAD9* locus, four novel SNPs annotating to *SMAD7* were reported (plus three established SNPs associated with *SMAD3*), all suggesting variation in inhibitory SMADs is likely of functional importance in human bone biology.^17^

The phenotype we describe here contrasts with that of activating mutations of the BMP receptor, *ACVR1*, which increase BMP signaling. However, in contrast to p.Leu22Pro *SMAD9* HBM, *ACVR1* mutations lead to a fatal condition, fibrous ossificans progressiva (FOP, OMIM#135100).^63^ In FOP, muscle tissue differentiates into bone after trivial injury, resulting in the formation of mature bone at multiple extraskeletal sites. *ACVR1* mutations may produce a more severe phenotype, compared with loss‐of‐function mutations in *SMAD9* reported here, since *ACVR1* also activates non‐SMAD‐dependent BMP signaling cascades, such as the NF‐κB and p38 MAP kinase (p38MAPK) pathways, which are upregulated in FOP *ACVR1* R206H monocytes.^64^

Given the benign phenotype observed in c.65T>C, p.Leu22Pro *SMAD9* carriers, our findings suggest that SMAD9 is worth consideration as a drug target for osteoporosis. Our zebrafish studies suggest that Smad9 is expressed in pre‐osteoblasts, consistent with the profile of an anabolic target capable of stimulating new bone formation through recruitment of early osteoblast progenitors. Given the pathological consequence of excess BMP activation in FOP, this pathway has not been prioritized as a possible therapeutic target in osteoporosis, despite the profound bone anabolic potential. Interestingly, phosphorylation of Smad9, as part of the Smad1/5/9 heterotrimer, has been researched in relation to fracture healing and bone regeneration: G‐protein‐coupled receptor kinase 2‐interacting protein‐1 (GIT1), a shuttle protein in osteoblasts, regulates Smad1/5/9 phosphorylation, which in turn mediates BMP2 regulation of Runx2 expression and thus endochondral bone formation at fracture sites.^65^, ^66^ Moreover, BMP has been administered locally to promote bone repair after surgery.^67^ Based on our findings, it is tempting to speculate that treatments suppressing SMAD9 activity might prove useful in treating osteoporosis, fractures, and possibly also sarcopenia. The potential pleiotropic association between one *SMAD9* variant and a composite cardiovascular phenotype represents the results of lifelong exposure to a variant rather than any potential short‐term perturbations in a gene pathway as might be exploited therapeutically.

Our study has limitations. All individuals with c.65T>C, p.Leu22Pro *SMAD9* HBM were female, reflecting the study design that favored those with a historical DXA scan who are more likely to be female. Whether findings will be similar in men is unknown, although no sex‐gene interaction has been described for the *LRP4*, *LRP5*, *LRP6*, or *SOST* sclerosing bone dysplasias. In the recent UK Biobank eBMD GWAS, LD score regression analyses suggested that the genetic architecture influencing male and female eBMD was largely shared with some significant differences between the sexes (rG = 0.91, SE = 0.012, *p* < 0.001),^17^ consistent with earlier epidemiological studies.^68^ The small sample of *SMAD9* HBM cases (*n* = 4 with pQCT) limited our ability to robustly evaluate associations statistically. The c.65T>C, p.Leu22Pro mutation is a reported SNP carried within the general population (eg, in the UK, an estimated 92,428 people might be expected to carry this mutation). This may be the case, given there is no indication that the phenotype affects reproductive fitness and HBM will not be overt unless a DXA scan is performed. Our GWAS was based on estimated heel BMD quantified by ultrasound rather than DXA‐measured BMD. Estimated heel BMD is not used routinely in clinical practice. However, we have previously demonstrated a strong overlap between genetic loci identified by eBMD GWAS and by DXA‐measured BMD GWAS.^44^

We report *SMAD9* as a novel HBM‐causing gene. The clinical phenotype of c.65T>C, p.Leu22Pro *SMAD9* HBM has many features in common with that of *LRP5* HBM but lacks the deleterious features that characterize *SOST* HBM (sclerosteosis). As reported for both *LRP5* and *SOST*, we demonstrate that a rare mutation in *SMAD9* is associated with an extreme bone phenotype and that common variation in *SMAD9* affects bone density within the general population. The role of SMAD9 in bone biology is supported by our finding of high levels of Smad9 expression in murine osteocytes and in skeletal elements of zebrafish larvae. Smad9 is thought to inhibit BMP signaling to reduce osteoblast activity; thus, we hypothesize *SMAD9* c.65T>C is a loss‐of‐function mutation reducing BMP inhibition, ultimately leading to enhanced bone formation. Our findings support SMAD9, and its role within the SMAD9‐dependent BMP signaling pathway, as a potential novel anabolic target for osteoporosis therapeutics that warrants further investigation.

## Disclosures

The authors declare that no competing interests exist.

## Supporting information


**File S1.** **Supplemental Methods**

**Movie S1.** 3D projection of confocal acquired z‐stack showing high Smad9 protein expression adjacent to osteoblasts in the larval zebrafish 6 day old opercle.

**Movie S2.** 2: 3‐dimensional rotating image of Fig. 6.

## References

[1] Wright NC , Looker AC , Saag KG , et al. The recent prevalence of osteoporosis and low bone mass in the United States based on bone mineral density at the femoral neck or lumbar spine. J Bone Miner Res. 2014;29(11):2520–6. 24771492 10.1002/jbmr.2269 PMC4757905

[2] Burge R , Dawson‐Hughes B , Solomon DH , Wong JB , King A , Tosteson A . Incidence and economic burden of osteoporosis‐related fractures in the United States, 2005‐2025. J Bone Miner Res. 2007;22(3):465–75. 17144789 10.1359/jbmr.061113

[3] McClung MR , Grauer A , Boonen S , et al. Romosozumab in postmenopausal women with low bone mineral density. N Engl J Med. 2014;370(5):412–20. 24382002 10.1056/NEJMoa1305224

[4] McClung MR , Brown JP , Diez‐Perez A , et al. Effects of 24 months of treatment with romosozumab followed by 12 months of denosumab or placebo in postmenopausal women with low bone mineral density: a randomized, double‐blind, phase 2, parallel group study. J Bone Miner Res. 2018;33(8):1397–406. 29694685 10.1002/jbmr.3452

[5] Brunkow ME , Gardner JC , Van Ness J , et al. Bone dysplasia sclerosteosis results from loss of the SOST gene product, a novel cystine knot‐containing protein. Am J Med Genet. 2001;68(3):577–89. 10.1086/318811 PMC1274471 11179006

[6] Staehling‐Hampton K , Proll S , Paeper BW , et al. A 52‐kb deletion in the SOST‐MEOX1 intergenic region on 17q12‐q21 is associated with van Buchem disease in the Dutch population. Am J Med Genet. 2002;110(2):144–52. 12116252 10.1002/ajmg.10401

[7] Little RD , Carulli JP , Del Mastro RG , et al. A mutation in the LDL receptor‐related protein 5 gene results in the autosomal dominant high‐bone‐mass trait. Am J Hum Genet. 2002;70(1):11–9. 11741193 10.1086/338450 PMC419982

[8] Whyte MP , McAlister WH , Zhang F , et al. New explanation for autosomal dominant high bone mass: Mutation of low‐density lipoprotein receptor‐related protein 6. Bone. 2019;127:228–43. 31085352 10.1016/j.bone.2019.05.003

[9] Gregson CL , Wheeler L , Hardcastle SA , et al. Mutations in known monogenic high bone mass loci only explain a small proportion of high bone mass cases. J Bone Miner Res. 2015;31(3):640–9. 26348019 10.1002/jbmr.2706 PMC4832273

[10] Gregson CL , Steel SA , O'Rourke KP , et al. “Sink or swim”: an evaluation of the clinical characteristics of individuals with high bone mass. Osteoporos Int. 2012;23(2):643–54. 21455762 10.1007/s00198-011-1603-4 PMC3261396

[11] Gregson CL , Newell F , Leo PJ , et al. Genome‐wide association study of extreme high bone mass: contribution of common genetic variation to extreme BMD phenotypes and potential novel BMD‐associated genes. Bone. 2018;114:62–71. 29883787 10.1016/j.bone.2018.06.001 PMC6086337

[12] Gregson CL , Sayers A , Lazar V , et al. The high bone mass phenotype is characterised by a combined cortical and trabecular bone phenotype: findings from a pQCT case–control study. Bone. 2013;52(1):380–8. 23103330 10.1016/j.bone.2012.10.021 PMC3526774

[13] Duncan EL , Danoy P , Kemp JP , et al. Genome‐wide association study using extreme truncate selection identifies novel genes affecting bone mineral density and fracture risk. PLoS Genet. 2011;7(4):e1001372. 21533022 10.1371/journal.pgen.1001372 PMC3080863

[14] McInerney‐Leo AM , Schmidts M , Cortes CR , et al. Short‐rib polydactyly and Jeune syndromes are caused by mutations in WDR60. Am J Hum Genet. 2013;93(3):515–23. 23910462 10.1016/j.ajhg.2013.06.022 PMC3769922

[15] Adzhubei IA , Schmidt S , Peshkin L , et al. A method and server for predicting damaging missense mutations. Nat Methods. 2010;7(4):248–9. 20354512 10.1038/nmeth0410-248 PMC2855889

[16] Kumar P , Henikoff S , Ng PC . Predicting the effects of coding non‐synonymous variants on protein function using the SIFT algorithm. Nat Protoc. 2009;4(7):1073–81. 19561590 10.1038/nprot.2009.86

[17] Morris JA , Kemp JP , Youlten SE , et al. An atlas of genetic influences on osteoporosis in humans and mice. Nat Genet. 2019;51:258–66. 30598549 10.1038/s41588-018-0302-x PMC6358485

[18] de Leeuw CA , Mooij JM , Heskes T , Posthuma D . MAGMA: generalized gene‐set analysis of GWAS data. PLoS Comput Biol. 2015;11(4):e1004219. 25885710 10.1371/journal.pcbi.1004219 PMC4401657

[19] Watanabe K , Stringer S , Frei O , et al. A global overview of pleiotropy and genetic architecture in complex traits. Nat Genet. 2019;51(9):1339–48. 31427789 10.1038/s41588-019-0481-0

[20] Hart T , Komori HK , LaMere S , Podshivalova K , Salomon DR . Finding the active genes in deep RNA‐seq gene expression studies. BMC Genomics. 2013;14:778. 24215113 10.1186/1471-2164-14-778 PMC3870982

[21] Youlten S , Baldock P , Leitch V , et al. Osteocytes express a unique transcriptome that underpins skeletal homeostasis. American Society for Bone and Mineral Research (ASBMR) 2017 Annual Meeting. Denver, CO;(LB‐1165)2017.

[22] Ferrer‐Costa C , Gelpí JL , Zamakola L , Parraga I , de la Cruz X , Orozco M . PMUT: a web‐based tool for the annotation of pathological mutations on proteins. Bioinformatics. 2005;21(14):3176–8. 15879453 10.1093/bioinformatics/bti486

[23] Schwarz JM , Rodelsperger C , Schuelke M , Seelow D . MutationTaster evaluates disease‐causing potential of sequence alterations. Nat Methods. 2010;7(8):575–6. 20676075 10.1038/nmeth0810-575

[24] Zimmermann L , Stephens A , Nam SZ , et al. A completely reimplemented MPI bioinformatics toolkit with a new HHpred server at its core. J Mol Biol. 2018;430(15):2237–43. 29258817 10.1016/j.jmb.2017.12.007

[25] Webb B , Sali A . Comparative protein structure modeling using modeller. Curr Protoc Bioinformatics. 2016;54:5.6.1–5.6.37. 27322406 10.1002/cpbi.3 PMC5031415

[26] Pettersen EF , Goddard TD , Huang CC , et al. UCSF Chimera—a visualization system for exploratory research and analysis. J Comput Chem. 2004;25(13):1605–12. 15264254 10.1002/jcc.20084

[27] Alexander C , Zuniga E , Blitz IL , et al. Combinatorial roles for BMPs and Endothelin 1 in patterning the dorsal‐ventral axis of the craniofacial skeleton. Development. 2011;138(23):5135–46. 22031543 10.1242/dev.067801 PMC3210495

[28] Spoorendonk KM , Peterson‐Maduro J , Renn J , et al. Retinoic acid and Cyp26b1 are critical regulators of osteogenesis in the axial skeleton. Development. 2008;135(22):3765–74. 18927155 10.1242/dev.024034

[29] Westerfield M . The zebrafish book. A guide for the laboratory use of zebrafish (Danio Rerio). Eugene, OR: University of Oregon Press; 2000.

[30] Bergen DJM , Stevenson NL , Skinner REH , Stephens DJ , Hammond CL . The Golgi matrix protein giantin is required for normal cilia function in zebrafish. Biol Open. 2017;6(8):1180–9. 28546340 10.1242/bio.025502 PMC5576078

[31] Hammond CL , Schulte‐Merker S . Two populations of endochondral osteoblasts with differential sensitivity to hedgehog signalling. Development. 2009;136(23):3991–4000. 19906866 10.1242/dev.042150

[32] Schindelin J , Arganda‐Carreras I , Frise E , et al. Fiji: an open‐source platform for biological‐image analysis. Nat Methods. 2012;9(7):676–82. 22743772 10.1038/nmeth.2019 PMC3855844

[33] Zheng HF , Forgetta V , Hsu YH , et al. Whole‐genome sequencing identifies EN1 as a determinant of bone density and fracture. Nature. 2015;526(7571):112–7. 26367794 10.1038/nature14878 PMC4755714

[34] Stitziel NO , Stirrups KE , Masca NG , et al. Coding variation in ANGPTL4, LPL, and SVEP1 and the risk of coronary disease. N Engl J Med. 2016;374(12):1134–44. 26934567 10.1056/NEJMoa1507652 PMC4850838

[35] Webb TR , Erdmann J , Stirrups KE , et al. Systematic evaluation of pleiotropy identifies 6 further loci associated with coronary artery disease. J Am Coll Cardiol. 2017;69(7):823–36. 28209224 10.1016/j.jacc.2016.11.056 PMC5314135

[36] Nelson CP , Goel A , Butterworth AS , et al. Association analyses based on false discovery rate implicate new loci for coronary artery disease. Nat Genet. 2017;49(9):1385–91. 28714975 10.1038/ng.3913

[37] Dallas SL , Bonewald LF . Dynamics of the transition from osteoblast to osteocyte. Ann N Y Acad Sci. 2010;1192:437–43. 20392270 10.1111/j.1749-6632.2009.05246.x PMC2981593

[38] Bergen DJM , Kague E , Hammond CL . Zebrafish as an emerging model for osteoporosis: a primary testing platform for screening new osteo‐active compounds. Front Endocrinol. 2019;10:6. 10.3389/fendo.2019.00006 PMC6361756 30761080

[39] Tsukamoto S , Mizuta T , Fujimoto M , et al. Smad9 is a new type of transcriptional regulator in bone morphogenetic protein signaling. Sci Rep. 2014;4:7596. 25534700 10.1038/srep07596 PMC4274517

[40] Butler WT , Mikulski A , Urist MR , Bridges G , Uyeno S . Noncollagenous proteins of a rat dentin matrix possessing bone morphogenetic activity. J Dent Res. 1977;56(3):228–32. 265954 10.1177/00220345770560030601

[41] Lowery JW , Rosen V . The BMP pathway and its inhibitors in the skeleton. Physiol Rev. 2018;98(4):2431–52. 30156494 10.1152/physrev.00028.2017

[42] Huang Z , Wang D , Ihida‐Stansbury K , Jones PL , Martin JF . Defective pulmonary vascular remodeling in Smad8 mutant mice. Hum Mol Genet. 2009;18(15):2791–801. 19419974 10.1093/hmg/ddp214 PMC2706683

[43] Arnold SJ , Maretto S , Islam A , Bikoff EK , Robertson EJ . Dose‐dependent Smad1, Smad5 and Smad8 signaling in the early mouse embryo. Dev Biol. 2006;296(1):104–18. 16765933 10.1016/j.ydbio.2006.04.442 PMC7116376

[44] Kemp JP , Morris JA , Medina‐Gomez C , et al. Identification of 153 new loci associated with heel bone mineral density and functional involvement of GPC6 in osteoporosis. Nat Genet. 2017;49(10):1468–75. 28869591 10.1038/ng.3949 PMC5621629

[45] Medina‐Gomez C , Kemp JP , Trajanoska K , et al. Life‐course genome‐wide association study meta‐analysis of total body BMD and assessment of age‐specific effects. Am J Hum Genet. 2018;102(1):88–102. 29304378 10.1016/j.ajhg.2017.12.005 PMC5777980

[46] Koay MA , Woon PY , Zhang Y , et al. Influence of LRP5 polymorphisms on normal variation in BMD. J Bone Miner Res. 2004;19(10):1619–27. 15355556 10.1359/JBMR.040704

[47] Uitterlinden AG , Arp PP , Paeper BW , et al. Polymorphisms in the sclerosteosis/van Buchem disease gene (SOST) region are associated with bone‐mineral density in elderly whites. Am J Hum Genet. 2004;75(6):1032–45. 15514891 10.1086/426458 PMC1182139

[48] Boyden LM , Mao J , Belsky J , et al. High bone density due to a mutation in LDL‐receptor‐related protein 5. N Engl J Med. 2002;346(20):1513–21. 12015390 10.1056/NEJMoa013444

[49] Hamersma H , Gardner J , Beighton P . The natural history of sclerosteosis. Clin Genet. 2003;63(3):192–7. 12694228 10.1034/j.1399-0004.2003.00036.x

[50] Xiao J , Purcell SA , Prado CM , Gonzalez MC . Fat mass to fat‐free mass ratio reference values from NHANES III using bioelectrical impedance analysis. Clin Nutr. 2018;37(6, Part A):2284–7. 29056283 10.1016/j.clnu.2017.09.021

[51] Frost M , Andersen T , Gossiel F , et al. Levels of serotonin, sclerostin, bone turnover markers as well as bone density and microarchitecture in patients with high‐bone‐mass phenotype due to a mutation in Lrp5. J Bone Miner Res. 2011;26(8):1721–8. 21351148 10.1002/jbmr.376

[52] Gregson CL , Poole KES , McCloskey EV , et al. Elevated circulating Sclerostin concentrations in individuals with high bone mass, with and without LRP5 mutations. J Clin Endocrinol Metab. 2014;99(8):2897–907. 24606091 10.1210/jc.2013-3958 PMC4207929

[53] Bonewald L . Use it or lose it to age: a review of bone and muscle communication. Bone. 2018;120:212–8. 30408611 10.1016/j.bone.2018.11.002 PMC6360108

[54] Tosca L , Brisset S , Petit FM , et al. Genotype‐phenotype correlation in 13q13.3‐q21.3 deletion. Eur J Med Genet. 2011;54(5):e489–e494. 21741501 10.1016/j.ejmg.2011.06.004

[55] Shintani M , Yagi H , Nakayama T , Saji T , Matsuoka R . A new nonsense mutation of SMAD8 associated with pulmonary arterial hypertension. J Med Genet. 2009;46(5):331–7. 19211612 10.1136/jmg.2008.062703

[56] Drake KM , Zygmunt D , Mavrakis L , et al. Altered MicroRNA processing in heritable pulmonary arterial hypertension: an important role for Smad‐8. Am J Respir Crit Care Med. 2011;184(12):1400–8. 21920918 10.1164/rccm.201106-1130OC PMC3262031

[57] Walcott BP , Winkler EA , Zhou S , et al. Identification of a rare BMP pathway mutation in a non‐syndromic human brain arteriovenous malformation via exome sequencing. Hum Genome Var. 2018;5:18001. 29844917 10.1038/hgv.2018.1 PMC5966745

[58] Ngeow J , Yu W , Yehia L , et al. Exome sequencing reveals germline SMAD9 mutation that reduces phosphatase and Tensin homolog expression and is associated with hamartomatous polyposis and gastrointestinal ganglioneuromas. Gastroenterology. 2015;149(4):886–9.e5. 26122142 10.1053/j.gastro.2015.06.027

[59] Le Goff C , Mahaut C , Wang Lauren W , et al. Mutations in the TGFβ binding‐protein‐like domain 5 of FBN1 are responsible for acromicric and geleophysic dysplasias. Am J Hum Genet. 2011;89(1):7–14. 21683322 10.1016/j.ajhg.2011.05.012 PMC3135800

[60] Loeys BL , Gerber EE , Riegert‐Johnson D , et al. Mutations in fibrillin‐1 cause congenital scleroderma: stiff skin syndrome. Sci Transl Med. 2010;2(23):23ra0. 10.1126/scitranslmed.3000488 PMC2953713 20375004

[61] Rommel K , Karck M , Haverich A , et al. Identification of 29 novel and nine recurrent fibrillin‐1 (FBN1) mutations and genotype‐phenotype correlations in 76 patients with Marfan syndrome. Hum Mutat. 2005;26(6):529–39. 16220557 10.1002/humu.20239

[62] Timberlake AT , Choi J , Zaidi S , et al. Two locus inheritance of non‐syndromic midline craniosynostosis via rare SMAD6 and common BMP2 alleles. Elife. 2016;5.pii:e20125. 10.7554/eLife.20125 PMC5045293 27606499

[63] Shore EM , Xu M , Feldman GJ , et al. A recurrent mutation in the BMP type I receptor ACVR1 causes inherited and sporadic fibrodysplasia ossificans progressiva. Nat Genet. 2006;38(5):525–7. 16642017 10.1038/ng1783

[64] Barruet E , Morales BM , Cain CJ , et al. NF‐κB/MAPK activation underlies ACVR1‐mediated inflammation in human heterotopic ossification. JCI Insight. 2018;3(22):e122958. 10.1172/jci.insight.122958 PMC6302947 30429363

[65] Sheu TJ , Zhou W , Fan J , et al. Decreased BMP2 signal in GIT1 knockout mice slows bone healing. Mol Cell Biochem. 2014;397(1):67–74. 25138700 10.1007/s11010-014-2173-5 PMC4418226

[66] Hankenson KD , Gagne K , Shaughnessy M . Extracellular signaling molecules to promote fracture healing and bone regeneration. Adv Drug Deliv. Rev. 2015;94:3–12. 26428617 10.1016/j.addr.2015.09.008

[67] Salazar VS , Gamer LW , Rosen V . BMP signalling in skeletal development, disease and repair. Nat Rev Endocrinol. 2016;12(4):203–21. 26893264 10.1038/nrendo.2016.12

[68] Duncan EL , Cardon LR , Sinsheimer JS , Wass JA , Brown MA . Site and gender specificity of inheritance of bone mineral density. J Bone Miner Res. 2003;18(8):1531–8. 12929944 10.1359/jbmr.2003.18.8.1531

