# Supplementary material for: A Rare Mutation in SMAD9 Associated With High Bone Mass Identifies the SMAD‐Dependent BMP Signaling Pathway as a Potential Anabolic Target for Osteoporosis
Source: J Bone Miner Res. 2019 Nov 14;35(1):92–105. doi: 10.1002/jbmr.3875 (PMC7004081; doi:10.1002/jbmr.3875)
Supplement: Supplementary file 1 — File S1. Supplemental Methods [file JBMR-35-92-s003.pdf]

Supplemental Methods

High Bone Mass cases from the UK

DXA databases containing 335,115 DXA scans were initially searched for individuals with a BMD T or Z-score ≥+4 at any site within the lumbar spine (LS) or hip, at 13 centres in England and Wales (9 Hologic, 4 Lunar). A further two centres with Hologic scanners contributed 23 similar individuals identified prospectively. All 1505 DXA images were visually inspected; 962 cases with significant osteoarthritis and/or other causes of raised BMD were excluded (*e.g*. surgical metalwork, Paget's disease, metastases (1)). Evidence of significant osteoarthritis (OA) on lumbar DXA scans is common. To reduce contamination of remaining DXA scans by more moderate OA, we refined case definition by restricting analyses to specific lumbar vertebra(e). At the largest centre, 562 scans with T/Z-score ≥+4 were graded for OA severity and examined in relation to BMD at lumbar vertebral levels (2, 3). In contrast to other lumbar vertebrae, L1 Z-score was not associated with OA, reflecting the recognized pattern of progressive OA changes seen in descending sequential lumbar vertebrae (4). Further, presence of high TH BMD did not correlate with co-existent LS OA.

As a generalized HBM trait should affect both spine and hip BMD, though not necessarily equally, HBM was defined as a) first Lumbar Vertebra (L1) Z-score of ≥ +3.2 plus Total Hip (TH) Z-score of ≥ +1.2 and/or b) TH Z-score ≥ +3.2 plus L1 Z-score of ≥ +1.2 (using age and gender-adjusted BMD Z-scores). A threshold of +3.2 was in keeping with the only published precedent for identifying HBM using DXA (5). Z rather than T-score was used to limit age bias. A standard deviation of +3.2 would be expected to identify a tail of 0.069% of a normal distribution (6). Of 533 unexplained HBM index cases invited to participate, 248 (47%) were recruited between September 2008 and April 2010 (7). Index cases were asked to pass on study invitations to their first-degree relatives and spouse/partner(s). These relatives and spouses were invited once; non-responders were not followed-up. Relatives/spouses with HBM were in turn asked to pass on study invitations to their first-degree relatives and spouses. Written informed consent was collected for all in line with the Declaration of Helsinki (8). Participants were excluded if under 18 years of age, pregnant or unable to provide written informed consent. All participants were assessed using a standardised structured history and examination questionnaire. DNA was extracted from peripheral venous blood using standard phenol/chloroform extraction techniques.

DXA measurements

Participants underwent DXA scanning (repeat in the case of index cases) using either GE Lunar Prodigy DXA (software version 13.2, GEHealthcare, Madison, WI, USA) or Hologic Discovery/W DXA (software version Apex 3.0, Hologic Inc. Bedford, MA, USA). Scans were acquired and analysed according to each manufacturer's standard scanning and positioning protocols as previously described (9). Total Body (TB) bone mineral content (BMC) and density (BMD), Fat Mass (FM) and Lean Mass (LM) were measured, together with L1 and TH BMD. All DXA images were reviewed for quality control purposes.

Peripheral Quantitative Computed Tomography (pQCT)

pQCT scans were performed at the distal and mid-shaft of the tibia (4 & 66% from the distal endplate) in the non-dominant lower limb using a Stratec XCT2000L (Stratec Medizintechnik, Pforzheim, Germany) as published previously (10). Cortical bone was defined using a threshold above 650mg/cm3 (optimal for bone geometry (11)). Trabecular bone was identified by elimination of cortical bone and therefore trabecular bone mineral density (tBMD) was defined as a density <650mg/cm3. Cortical thickness, periosteal circumference and endosteal circumference were derived using a circular ring model. Further cortical parameters were measured: cortical bone mineral density (cBMD), total bone area (TBA) (i.e. total bone cross-section, reflecting periosteal expansion), cortical bone area (CBA) (reflecting a combination of periosteal and endosteal expansion) and CBA/TBA (%). Strength Strain Index (SSI) was calculated according to Stratec's user manual (SSI= SM*(cBMD[mg/cm3]/ 1200[mg/cm3]), where 1200 mg/cm3 represents the normal physiological density of bone (stated by Stratec) and SM (Section Modulus)=CSMI/ periosteal radius, where CSMI (Cross-Sectional Moment of Inertia [cm4])=Π(periosteal radius4-endosteal radius4)/4) (12).

Twenty controls were scanned twice after repositioning; measurement precision (CV) was 1 to 3% [11]. Stratec pQCT machines were calibrated using a COMAC phantom; mean (SD) difference between scanners was 1.18 (0.82)%.

Blood testing including bone turnover markers and DNA extraction

Two non-fasted EDTA samples were collected and serum separated and frozen within 4 hours to -80°C. Bone formation (Procollagen type 1 amino-terminal propeptide [P1NP], total osteocalcin) and resorption (β-C-telopeptides of type I collagen [βCTX]) markers were measured by Electrochemiluminescence immunoassays (ECLIA) performed on the COBAS e601 analyser (Roche Diagnostics, Burgess Hill, UK). Inter-assay coefficient of variation (CV) for P1NP was <3.0% across the range 5-1200 µg/L; osteocalcin was <5.0% across the range 0.5-300 µg/L; βCTX was <3.0% across the range 0.01-6 µg/L. Sclerostin was measured using an enzyme-linked immunosorbent assay (ELISA) kit BI-20492 (Biomedica GmbH, Vienna, Austria) with assay CV <9.0% across the range 2.6-240 pmol/L. DNA was extracted from peripheral venous blood using standard phenol/ chloroform extraction.

Ethical Approvals

Written informed consent was collected for all participants in line with the Declaration of Helsinki (8). The UK HBM study was approved by the Bath Multi-centre Research Ethics Committee (REC: 05/Q2001/78) and at each NHS Local REC.

The AOGC study was approved by the Queensland Office of Human Research Ethics Committee (Ref:2008/018), the University of Queensland (Ref:200800376) and/or relevant research ethics authorities at each participating center. Directly recruited participants gave written, informed consent. Some participants were recruited through genetic and/or clinical studies (all with ethical approval) but also provided written informed consent to contribute to collaborative genetic studies (13-15). DNA was obtained from peripheral venous blood or from saliva through standard methods.

Whole exome sequencing

Sequencing libraries for 859 samples (240 UK HBM, 126 AOGC HBM, 493 AOGC LBM) were constructed in two batches, determined by sample availability, the first (85 HBM, 619 AOGC) using the Illumina TruSeqDNA sample preparation kit, combined in pools of six for target capture by the Illumina TruSeq Exome v2.0 Enrichment Kit (64Mb capture) and assessed pre and post-capture for quality and yield with the Agilent High Sensitivity DNA assay and KAPA Library Quantification Kit. For the second batch (155 HBM), exome capture was performed using the Nextera® Rapid Capture Exome Enrichment Kit (Cat No FC-140-1003) (62Mb capture) (Illumina, San Diego, California, USA) as per the Nextera® Rapid Capture Enrichment. In both cases massive parallel sequencing was performed with six samples per flow cell lane on the Illumina HiSeq2000 platform and version 2 SBS reagents to generate 100 bp paired-end reads.

Base calling, sequence alignment and variant calling were all performed as previously described (16). In brief, after demultiplexing, the Illumina Data Analysis Pipeline software (CASAVA v.1.8.2) was used for initial base calling. Sequence data were aligned to the current build of the human genome (UCSC Genome Browser, hg19, released February 2009) via the Novoalign alignment tool (v.2.08.02 1) (17); sequence alignment files were converted by SAMtools (v.0.1.14) (18) and Picard tools (v.1.42). SNPs and indels were called with the Genome Analysis Toolkit (GATK v.5506) (19, 20) and annotated by ANNOVAR (21). Further analysis of sequence data was performed with custom R and Bioconductor scripts. Good-quality SNPs, excluding those with a genotype quality score <60 were retained (determined by the GATK algorithm; range 0 [poor] to 100 [excellent]). Platform related artefacts were identified as variants where the allele count was 6 SDs higher than that expected from the maximum population minor allele frequency (MAF) under the binomial approximation. Remaining SNPs and indels were assessed according to prediction of potentially damaging consequence (‘‘nonsynonymous SNV,’’ ‘‘splicing,’’ ‘‘frameshift substitution,’’ ‘‘stopgain SNV,’’ ‘‘stoploss SNV’’) by using both RefSeq and UCSC transcripts. Further filtering excluded SNPs with a MAF <0.05 (observed in NCBI dbSNP (GRCh37/ Hg19), 1000 Genomes (22), ExAC (http://exac.broadinstitute.org/), or internal databases from >3000 exomes), 1000 Genomes small indels (called with DINDEL (23)). Variants not present in any database were considered novel. Genotype Quality scores for the two additional isolated HBM cases were 99 and read depths were 15,14 and 21,19.

Sanger sequencing validation of pedigree based HBM mutation

Polymerase chain reaction (PCR) amplification of identified exons was performed on 50ng genomic DNA in a reaction mix consisting; 10X Immolase reaction buffer, 10mM dNTPs, 50mM $MgCl_2$, 5µM each primer, 0.5 U Immolase polymerase Taq (Bioline Reagents Ltd, London), and water to final volume of 25µl. PCR cycling conditions and primer sequences are shown below. Samples were Sanger sequenced using standard techniques (BigDye v3.1 chemistry, Life Technologies Corporation, California), and capillary sequenced (3130 Genetic Analyzer, Life Technologies Corporation, California).

SMAD9_Ex2 Primers

Fwd primer: CACCCTGTTCAAGGGCTTAG

Rvs primer: CGCGACAGTAAATCACATGG

PCR Reaction:

dH_2O (PCR grade)	12.2
10x ImmoBuffer	2.5
dNTPs<br>(2.5 mM each dNTP, 10 mM total dNTPs)	2.5
$MgCl_2$ (50 mM)	0.75
F (5 µM)	1
R (5 µM)	1
Immolase Taq (5 U / µL)	0.05
DNA template (10 ng /µL) (50ng total)	5
**Total**	**25**

PCR Cycling conditions:

1. 96˚C for 10 minutes
2. 96˚C for 45 seconds
3. 60˚C for 45 seconds
4. 72˚C for 60 seconds

Go to step 2, 34 more times (35 cycles)

5. 72˚C for 10 minutes
6. 4°C Hold

Multi-marker Analysis of GenoMic Annotation (MAGMA) in UK Biobank

Gene-based tests of association were performed on 362,924 unrelated white British subjects (54% female, GCTA-GRM derived pairwise relatedness <0.10) from the UK Biobank study that had valid quantitative ultrasound derived heel eBMD and high quality genome-wide HRC and 1000G/UK10K imputed data from the January 2018 release [*i.e.* 20,490,436 genetic variants with an information quality score >0.3, MAF >0.05, minor allele count >5, genotyping hard call rate >0.95, and weak evidence of deviation from Hardy-Weinberg equilibrium ($p > 1x10^{-6}$)]. Detailed methodology is published elsewhere (24). Gene-based tests of association were implemented in MAGMA v1.06 (25) using a multi-model approach combining association results from three separate gene analysis models: principal components regression, SNP-wise Mean $\chi 2$ model [*i.e.* test statistic derived as sum of -log(SNP p-value)] and SNP-wise Top $\chi 2$ model [(test statistic derived as sum of -log(SNP p-value) for top SNPs)] to produce an aggregate p-value corresponding to the association between each of the 19,361 protein coding genes (+/- 20kb) and BMD, adjusting for age, sex, genotyping array, assessment center and 10 ancestry informative principal components. Statistics for all genes within +/-800kb of *SMAD9* and *CHNR1* were looked-up.
Regional association plots were generated using LocusZoom (v1.3) (26) in conjunction with summary association results from Morris *et al* 2018 (24).

Supplemental Results 1: Clinical phenotypes

III.1: Index Case (c.65T>C, p.Leu22Pro)

The 33-year-old index case, with BMD Z-Scores +3.2 at the total hip and +4.5 at L1, had only sustained one traumatic fracture aged 20 months. She reported lower leg and ankle pain. Other than myopia corrected by glasses, she had no visual or auditory impairments, no significant dental history, no back pain or neuropathy, and no pulmonary disease. She had a history of bipolar disorder, polycystic ovary syndrome (PCOS) and psoriasis (no joint involvement), with corresponding medications. Menstrual disturbance secondary to PCOS had prompted her original DXA referral. She had large feet (shoe size UK 10, Euro 42-43, US 12). She was tall and obese (BMI 43.6), with a broad frame, enlarged mandible and a 4mm torus mandibularis. She had normal joints and no evidence of nerve impingement.

II.2: Mother of the index case (c.65T>C, p.Leu22Pro)

The 55-year-old mother, with BMD Z-Scores +3.3 at the total hip and at L1, had never sustained a fracture. Six years earlier she had had a right calcaneal spur surgically removed. She had widespread joint pains affecting ankles, knees (with previous arthroscopy), hips, shoulders, hands and feet, limiting mobility to 50 yards, with a diagnosis of fibromyalgia prompting high analgesic use. She had a history of asthma, hypertension, hypercholesterolaemia and depression with corresponding medications, with normal menopause at 53. She had no visual or auditory impairments, or dental history of note. She was tall and obese (BMI 41.4), with above average shoe size, a broad frame, enlarged mandible but no tori. She had a full range of movement in all joints, bilateral knee crepitus, bilateral pes planus and no signs of nerve compression.

III.2: Half-sister to index case (c.65T>C, p.Leu22Pro)

The 22-year-old half-sister, with BMD Z-Scores +4.8 at the total hip and +2.6 at L1, had not fractured. She had had sciatica for five years, lumbar back pain and fronto-temporal headaches for 11 years, with a diagnosis of migraine. She had no significant dental history, no visual or auditory impairments, but was unable to float. She had had a normal menarche at 12. She used inhalers for asthma and medication for depression and anxiety. She was tall and obese (BMI 43), with above average shoe size, a broad frame, enlarged mandible, a torus palatinus in the midline of her hard palate (3cm x 7mm), normal joint movement and no signs of nerve compression.

I.2: Grandmother of index case (wild-type)

The 75-years-old grandmother, who did not have HBM (BMD Z-Scores +0.1 at the total hip and +0.8 at L1) had never sustained a fracture. She had osteoarthritis affecting knees, hips, lumbar spine, fingers, left elbow, limiting mobility to 3 metres and a wheelchair. She had had auditory impairment since school and had had cataracts removed. She was overweight (BMI 28.1) with normal shoe size, a normal frame, mandible and no tori, with reduced extension of the right elbow and left knee, with bilateral crepitus of her knees. She died aged 81 of pneumonia secondary to advanced Emphysema.

Isolated HBM case (c.65T>C, p.Leu22Pro) from the UK

This 55-year-old female, with BMD Z-Scores +5.0 at the total hip and +4.7 at L1, had never fractured and reported difficulty floating. Her adult left upper cuspid tooth had never erupted; wisdom teeth had been extracted for overcrowding. She had noticed her own mandible enlargement. She had a congenital astigmatism of her left eye with poor vision, and congenital bilateral pes planus. She had no auditory impairment. She took tamoxifen post-surgery for breast cancer. She was obese (BMI 35) with a broad frame, mandible enlargement, but no tori. She had normal joints and no signs of nerve compression.

Fat mass index and Fat-free mass index were calculated as below:

	HBM Pedigree					Additional Isolated HBM cases	
	UK III.1	UK III.2	UK II.2	UK I.2		UK case	Australian case
***SMAD9* Mutation**	Leu22Pro	Leu22Pro	Leu22Pro	WT		Leu22Pro	Leu22Pro
Height (m)	1.78	1.73	1.75	1.61		1.60	1.62
Fat mass (kg)	73.2	64.5	64.8	25.4		34.0	-
Lean mass (kg)	61.3	65.6	59.5	45		52.4	-
Fat mass index (FMI)	23.1	21.5	21.2	9.9		13.3	-
No. SDs FMI is from normal	+3.39	+2.98	+2.90	+0.07		+0.93	-
Fat-free mass index (FFMI)	19.4	21.8	19.4	17.5		20.5	-
No. SDs FFMI is from normal	+1.20	+2.34	+1.24	+0.34		+1.72	-

FMI = Fat mass (kg)/height m^2.
NHANES III mean (SD) FMI for adult women = 9.65 (4.1) (27).
FFMI = Fat-free (lean) mass (kg)/height m^2.
NHANES III mean (SD) FFMI for adult women = 16.71 (2.19) (27).

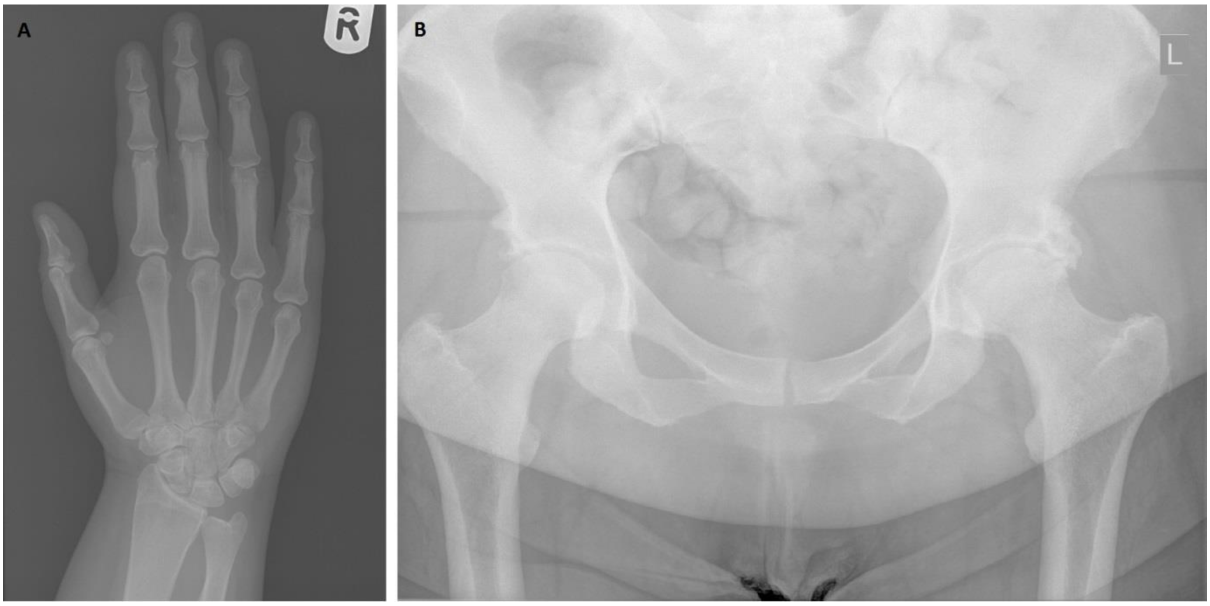

Supplemental Figure 1. Plain Radiographs of the (A) right hand and (B) pelvis from *III.1*: Index Case

(A) shows prominent metacarpal and proximal phalangeal cortical thicknesses. (B) shows generalised increased density within the iliac blades and thick proximal femoral cortices but no expansive bone changes and normal trabeculation. Bilateral hip joint degenerative changes and new bone formation at the anterior inferior iliac spines bilaterally

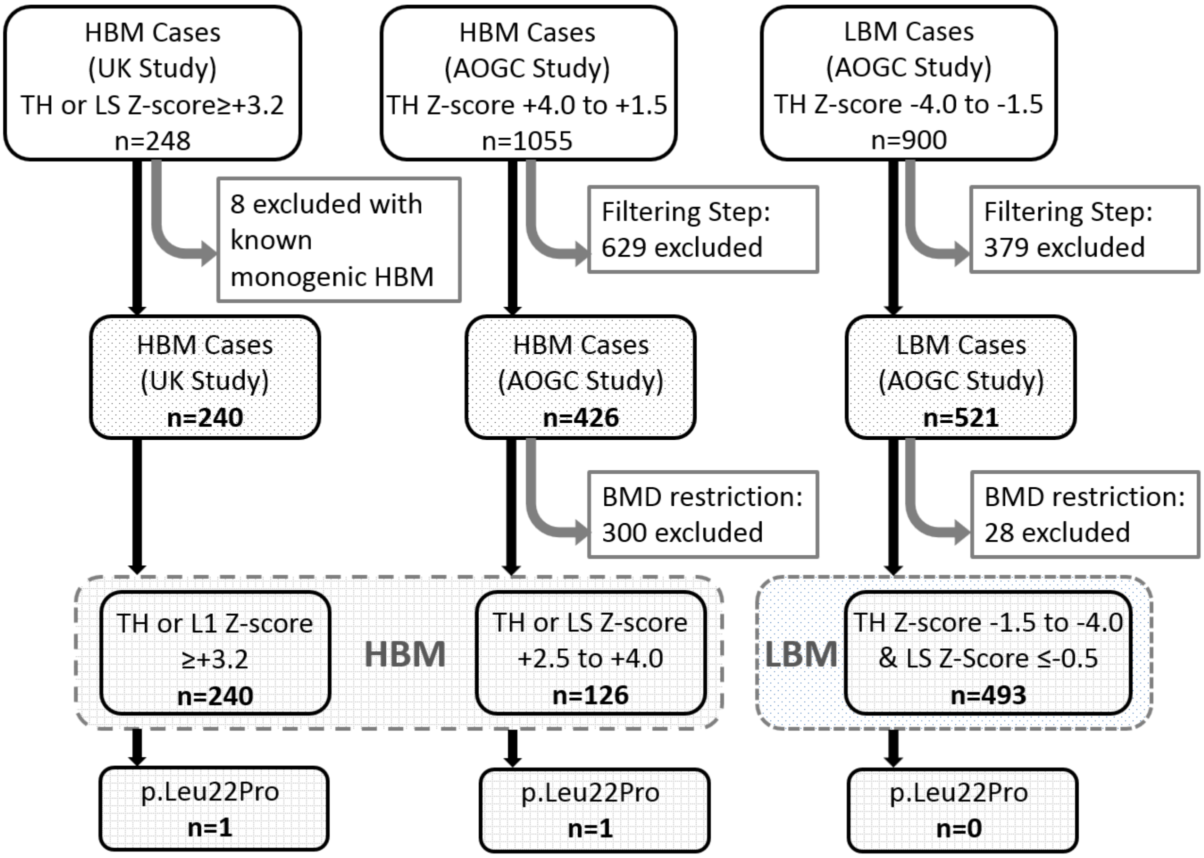

Supplemental Figure 2. Flow diagram explaining how further HBM cases were identified for whole-exome sequencing

TH; Total Hip. LS; Lumbar Spine. QC; Quality Control.
Filtering step restricted sample to individuals who were unrelated, Caucasian ancestry, with complete weight and height data, enough high-quality DNA available for WES, and were able to be sequenced within our financial constraints. BMD restriction ensured all HBM cases had TH or LS Z-score ≥+2.5, and all LBM Cases a TH Z-Score < -1.5 and LS Z-Score ≤-0.5.

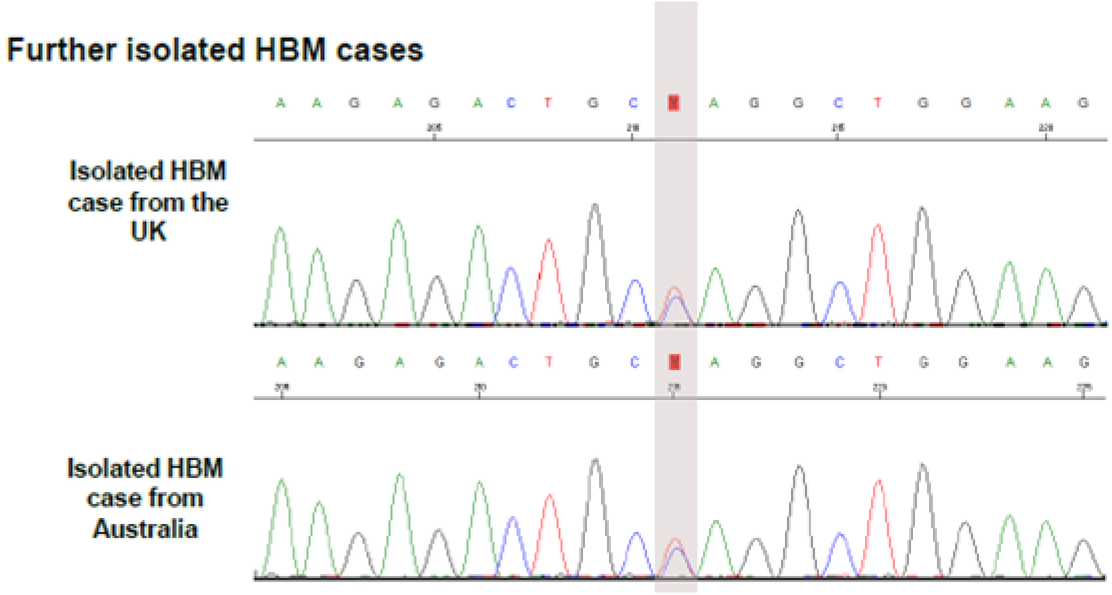

Supplemental Figure 3. Electrophoretograms for the additional two isolated unrelated HBM cases with a *SMAD9* c.65T>C, p.Leu22Pro mutation

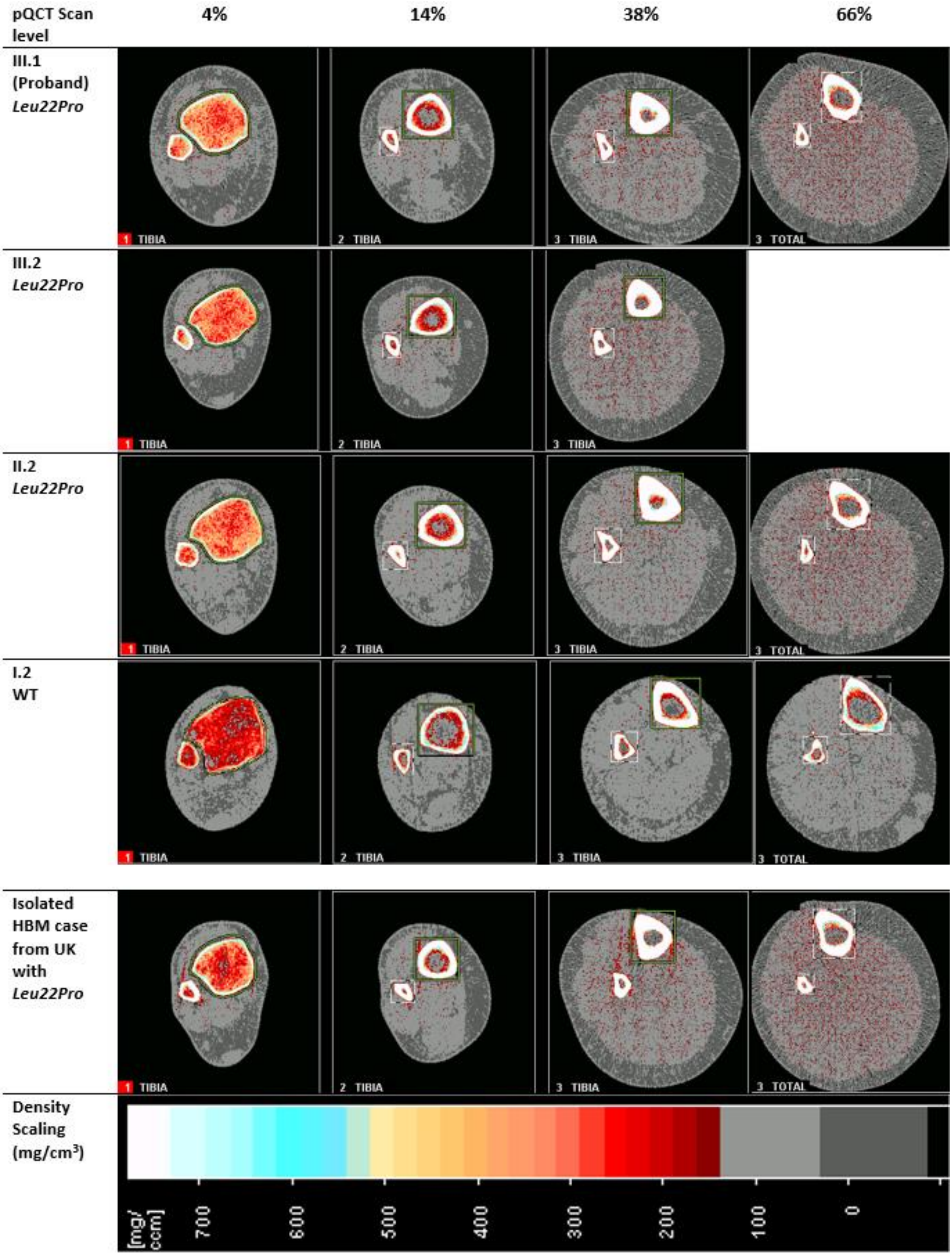

Supplemental Figure 4: Tibia pQCT scan results from HBM pedigree and isolated HBM case with *Leu22Pro* mutation. Images from sequential pQCT scans taken at 4, 14, 38 and 66% along the tibia from the distal endplate. The 4% site shows predominantly trabecular bone, whilst cortical bone is thickest at the 38% site. The 66% site shows muscle size; of note *III.2* had too large a calf to fit within the pQCT gantry and hence this image was not attained.

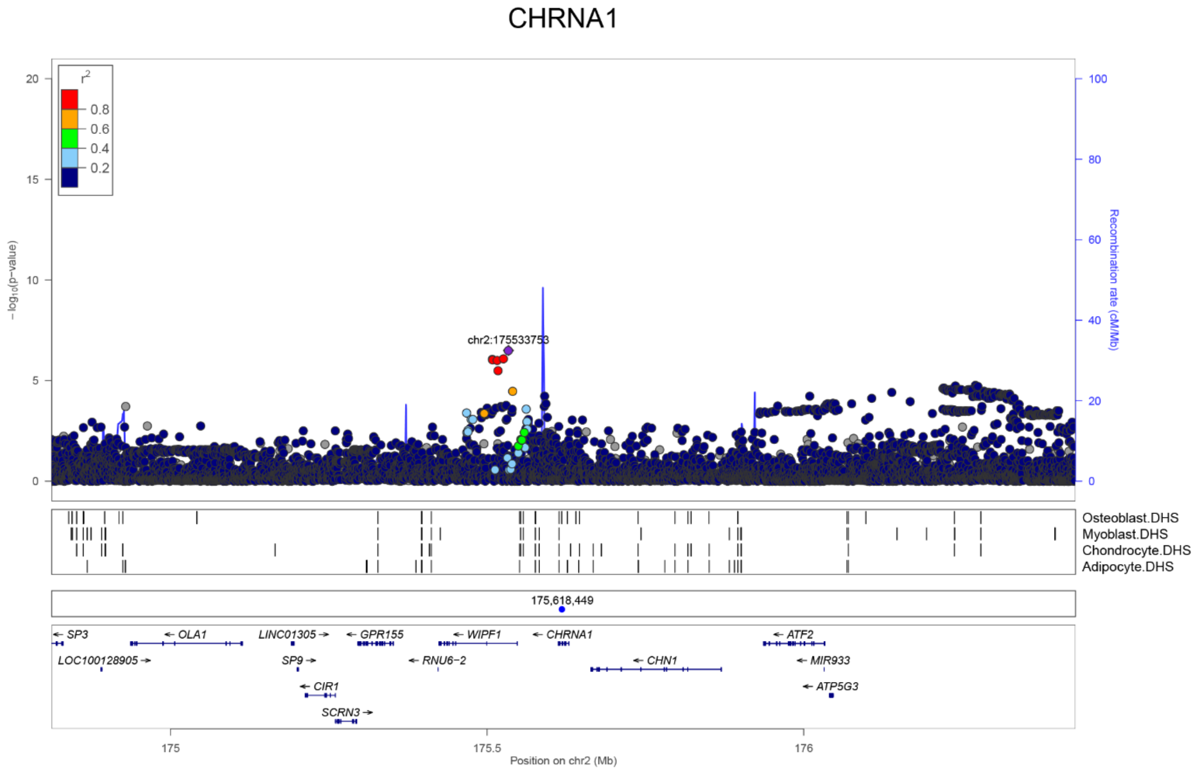

Supplemental Figure 5. GWAS for eBMD measured in UK Biobank: Regional association plot for the locus containing *CHRNA1*

Top panel: circles show unconditioned GWAS *P*-values and genomic locations of imputed SNPs within +/- 800kb of the 5' and 3' UTR of each gene. Different colours indicate varying degrees of pairwise linkage disequilibrium between the sentinel eBMD associated SNP (purple diamond) and all other SNPs. Second panel: Vertical shaded areas correspond to locations of DNase I hypersensitive sites (DHSs) characteristic of: skeletal muscle myoblasts cell line (E120), osteoblast primary cells (E129), mesenchymal stem cell derived chondrocyte cultured cells (E049) and mesenchymal stem cell derived adipocyte cultured cells (E023). Red shading depicts intersections between DHSs and genome-wide significant SNPs. Black shading denotes instances in which any other SNPs intersect DHSs. Third panel: Blue circle shows the position of the putative causal mutation c.560T>C, p.Leu187Pro. Fifth panel: Horizontal lines represent genes with vertical lines annotating the location of exons. Arrows indicate the direction in which each gene is transcribed.

A

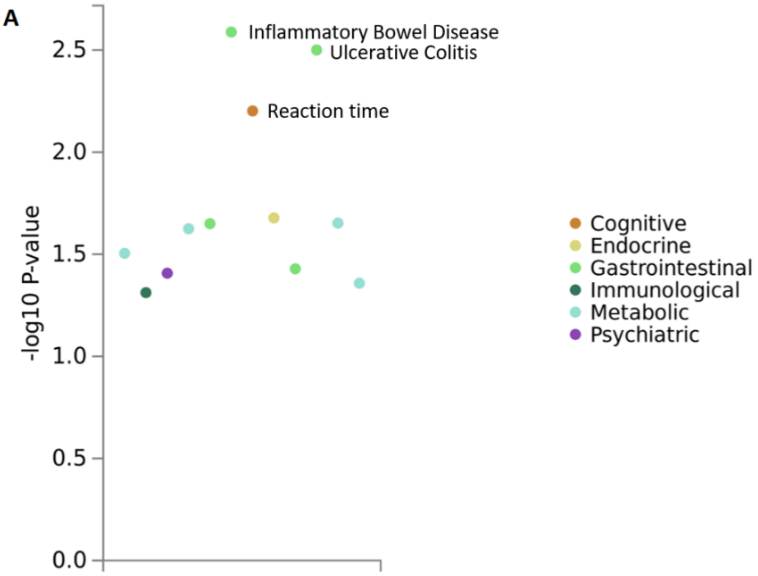
-log10 P-value
2.5
2.0
1.5
1.0
0.5
0.0
Inflammatory Bowel Disease
Ulcerative Colitis
Reaction time
Cognitive
Endocrine
Gastrointestinal
Immunological
Metabolic
Psychiatric

B

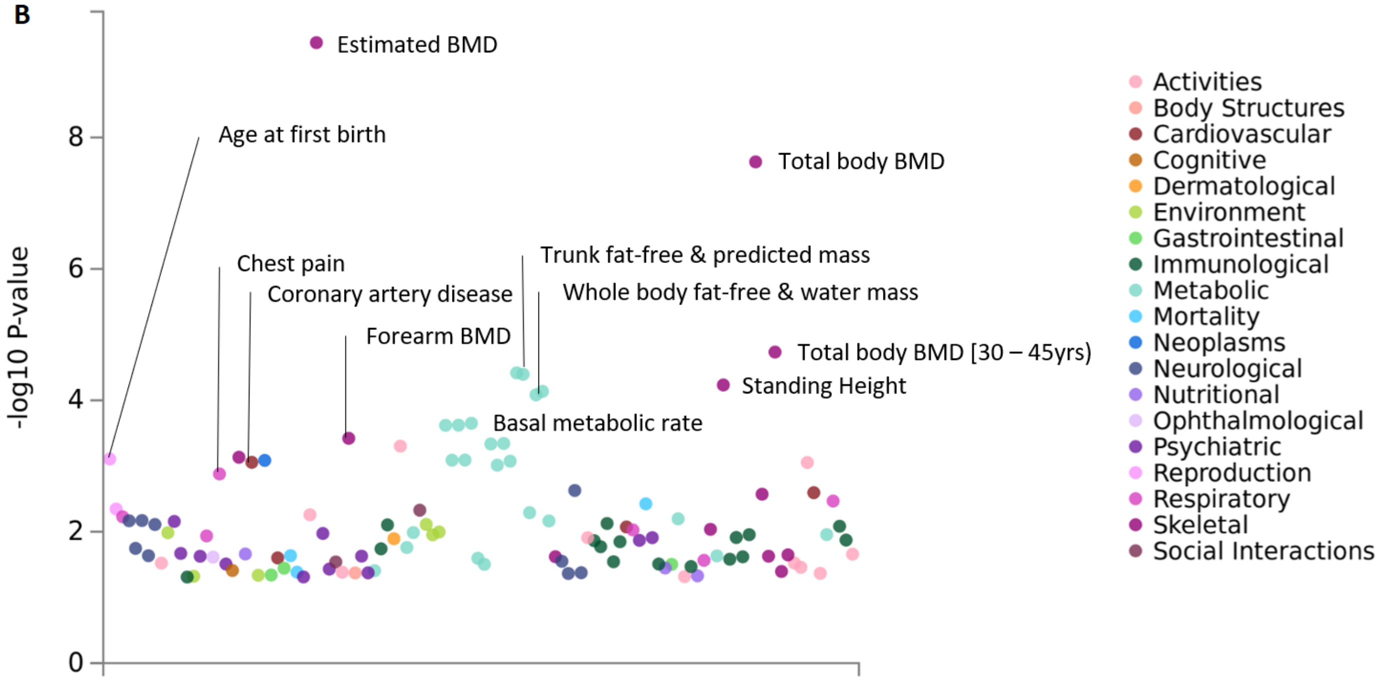
-log10 P-value
8
6
4
2
0
Estimated BMD
Age at first birth
Total body BMD
Chest pain
Coronary artery disease
Trunk fat-free & predicted mass
Whole body fat-free & water mass
Forearm BMD
Total body BMD [30 – 45yrs)
Standing Height
Basal metabolic rate
Activities
Body Structures
Cardiovascular
Cognitive
Dermatological
Environment
Gastrointestinal
Immunological
Metabolic
Mortality
Neoplasms
Neurological
Nutritional
Ophthalmological
Psychiatric
Reproduction
Respiratory
Skeletal
Social Interactions

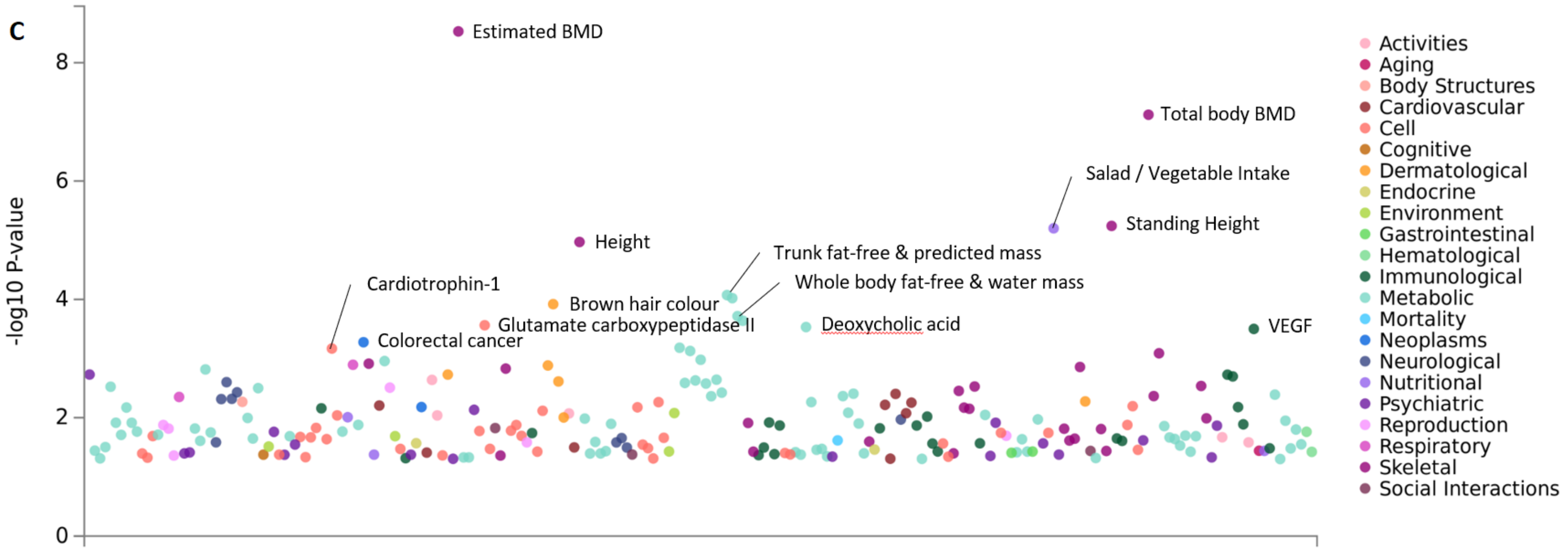

Supplemental Figure 6. PheWAS for:
A) The HBM variant (rs111748421)
B) The UK-Biobank Study eBMD GWAS variant (rs12427846)
C) The *SMAD9* gene, using on gene-based test of association derived by MAGMA

(x-axis is ordered alphabetically)

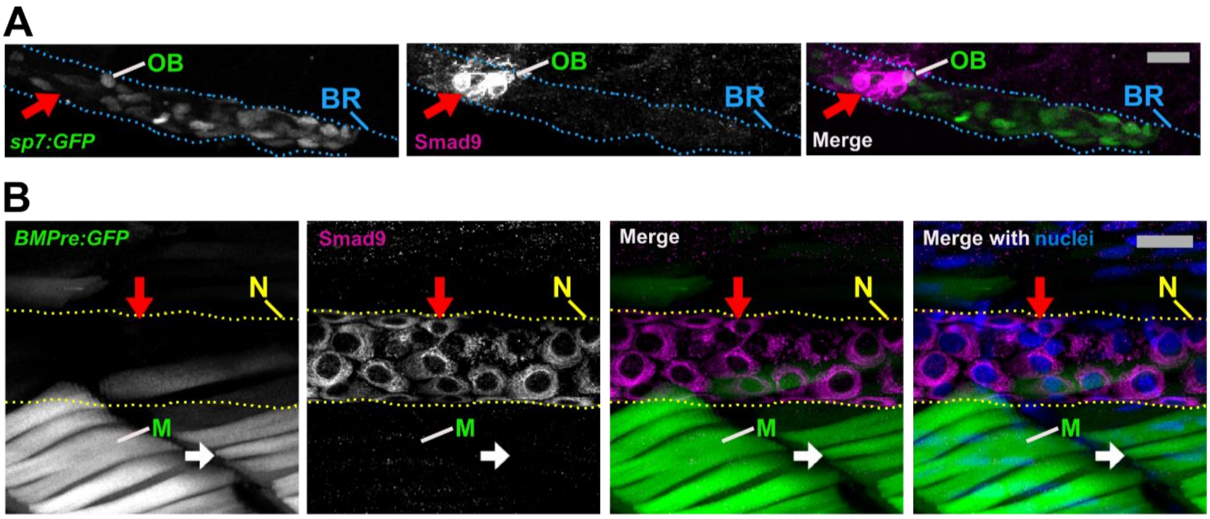

Supplemental Figure 7. Smad9 is highly expressed adjacent to osteoblasts in the juvenile branchiostegal ray and in the developing notochordal sheath.
A) High Smad9 expression (magenta, red arrow) in the anterior region of the *sp7:GFP* osteoblast (green) decorated branchiostegal ray (red arrow, intramembranous bone) at 6 days post fertilisation (dpf). Smad9 expression is observed adjacent to *sp7* promoter-driven GFP positive cells (osteoblasts). Blue dotted line outlines the branchiostegal ray.
B) At 7 dpf, strong cytoplasmic Smad9 expression was seen in notochordal sheath cells (magenta, red arrow, nucleus DAPI staining in blue) that cover the notochord (yellow dotted line) and which will contribute to vertebrae formation in late larval stages. Smad9-negative and BMP reporter (BMPre) GFP-positive muscle fibres were observed in the trunk muscles adjacent to the cloaca (green, white arrow).

A-B: scale bar is 20 µm, pictures in anterior-posterior (left-right) and dorsal-ventral (top-bottom) orientation from a lateral view, and all maximum intensity z-projection confocal images. Abbreviations: BR, branchiostegal ray; DAPI, 4′,6-diamidino-2-phenylindole; M, muscle; N, notochord; OB, osteoblast.

Supplemental Movie 1. 3D projection of confocal acquired z-stack showing high Smad9 protein expression adjacent to osteoblasts in the larval zebrafish 6 day old opercle. Smad9 in magenta and *sp7:GFP* positive osteoblasts in green. 360° rotation rotating at 6° per frame (from Figure 4C image). Note that Smad9 is distinctly separated from the GFP staining.

Supplemental Movie 2: 3-dimensional rotating image of Fig. 6

Supplemental Table 1: Characteristics measured by pQCT of the p.Leu22Pro *SMAD9* HBM pedigree members and two further isolated p.Leu22Pro *SMAD9* HBM individuals

	HBM Pedigree					Additional Isolated HBM cases	
	UK III.1	UK III.2	UK II.2	UK I.2		UK case	Australian case
***SMAD9* Mutation**	Leu22Pro	Leu22Pro	Leu22Pro	WT		Leu22Pro	Leu22Pro
Age	33	22	55	75		55	57
**pQCT 4% Distal Tibia**							
Total Bone Area (mm^2)	1051	1228	1051	1118		922	-
Trabecular BMD (mg/cm^3)	361	334	341	269		333	-
Cortical thickness (mm)	3.29	1.90	1.59	0.02		1.72	-
**pQCT 66% Mid-shaft Tibia**							
Total Bone Area (mm^2)	603	n/a	610	530		612	-
Cortical BMD (mg/cm^3)	1159	n/a	1151	1025		1139	-
Cortical thickness (mm)	4.85	n/a	4.93	2.15		5.10	-
Cortical Bone Area (mm^2)	348	n/a	355	161		366	-
Cortical/Total Bone Area (%)	57.7	n/a	58.3	30.4		59.7	-
SSI (mm^3)	1658	n/a	1682	756		1700	-
Muscle area (mm^2)	7956	n/a	8098	4889		8948	-

HBM: High Bone Mass, BMD: Bone Mineral Density, SD: Standard Deviation, SSI: Strength Strain Index, WT: Wildtype. n/a, not available as the leg was too large to fit into the pQCT gantry

Supplemental Table 2: Multi-marker Analysis of GenoMic Annotation (MAGMA) in UK Biobank; summary statistics for all genes within +/- 800kb of *SMAD9*

GENE ID	ENTREZ ID	CHR	START POSITION	STOP POSITION	No. of SNPs[a]	No. of PARAM[b]	Z STAT[c]	P_SNPWISE_MEAN[d]	P_SNPWISE_TOP1[e]	P_PCREG[f]	P_JOINT[g]
SOHLH2	54937	13	36722345	36808752	636	162	1.3622	2.10E-01	4.52E-01	2.86E-02	8.66E-02
CCDC169-SOHLH2	100526761	13	36722345	36891992	1296	198	1.2181	1.13E-01	4.96E-01	7.71E-02	1.12E-01
CCDC169	728591	13	36781179	36891992	872	142	0.8052	9.99E-02	7.56E-01	1.82E-01	2.10E-01
SPG20	23111	13	36855775	36964317	713	175	1.9677	1.13E-02	8.92E-02	2.78E-01	2.46E-02
CCNA1	8900	13	36985257	37037019	321	134	1.1017	9.32E-02	9.00E-02	6.76E-01	1.35E-01
SERTM1	400120	13	37228049	37291976	375	158	-0.1693	7.64E-01	4.81E-01	3.27E-01	5.67E-01
RFXAP	5994	13	37373339	37423740	337	134	-0.36054	6.54E-01	5.47E-01	4.08E-01	6.41E-01
SMAD9	***4093***	***13***	***37398968***	***37514409***	***833***	***268***	***8.2496***	***6.93E-11***	***6.65E-11***	***1.75E-02***	***7.94E-17***
ALG5	29880	13	37503907	37593504	533	182	1.4424	4.83E-01	7.60E-03	5.50E-01	7.46E-02
EXOSC8	11340	13	37554678	37603751	254	116	1.8791	2.53E-01	2.77E-03	4.38E-01	3.01E-02
SUPT20H	55578	13	37563449	37653850	469	156	1.8396	2.10E-01	6.98E-03	4.07E-01	3.29E-02
CSNK1A1L	122011	13	37657397	37699801	301	88	-0.021843	5.59E-01	3.31E-01	4.33E-01	5.09E-01
POSTN	10631	13	38116719	38192981	593	151	0.39879	3.20E-01	1.98E-01	5.45E-01	3.45E-01

Using data from 362924 unrelated white British UK Biobank participants.
[a] The number of SNPs annotated to the gene
[b] The number of parameters used in the model
[c] The Z-value for the gene, based on its (permutation) p-value
[d] P-value derived from SNP-wise mean $\chi 2$ model
[e] P-value derived from SNP-wise top $\chi 2$ model
[f] P-value derived from principal components linear regression model
[g] Aggregate p-value derived from all three methods above (*i.e.* d – f)
Gene-based significance threshold ($p<2.87x10^{-6}$) (24)

Supplemental Table 3: Multi-marker Analysis of GenoMic Annotation (MAGMA) in UK Biobank; summary statistics for all genes within +/- 800kb of *CHRNA1*

GENE ID	ENTREZ ID	CHR	START POSITION	STOP POSITION	No. of SNPs[a]	No. of PARAM[b]	Z STAT[c]	P_SNPWISE_MEAN[d]	P_SNPWISE_TOP1[e]	P_PCREG[f]	P_JOINT[g]
SP3	6670	2	174751187	174850430	797	207	0.7028	1.98E-01	6.34E-01	1.36E-01	2.41E-01
LOC100128905	100128905	2	174870014	174911886	275	111	2.6817	1.39E-03	4.01E-01	2.39E-02	3.66E-03
OLA1	29789	2	174917175	175133365	1375	211	-0.54926	5.00E-01	6.61E-01	5.41E-01	7.09E-01
SP9	100131390	2	175179769	175225859	305	116	-0.96924	9.23E-01	6.16E-01	5.90E-01	8.34E-01
CIR1	9541	2	175192878	175280443	519	152	-1.0406	8.16E-01	7.63E-01	5.27E-01	8.51E-01
SCRN3	79634	2	175240457	175314303	475	122	-0.88671	6.44E-01	7.47E-01	6.32E-01	8.12E-01
GPR155	151556	2	175276299	175371816	562	171	0.022405	5.05E-01	4.71E-01	3.31E-01	4.91E-01
WIPF1	7456	2	175404302	175567667	847	255	2.5592	1.28E-03	1.38E-01	1.08E-01	5.25E-03
CHRNA1	**1134**	**2**	**175592320**	**175649200**	**380**	**116**	**3.0694**	**3.98E-03**	**6.32E-02**	**4.94E-03**	**1.07E-03**
CHN1	1123	2	175644042	175890671	1320	292	1.0285	1.35E-01	7.73E-01	7.36E-02	1.52E-01
ATF2	1386	2	175916978	176052934	887	192	0.45519	2.47E-01	5.37E-01	2.29E-01	3.24E-01
ATP5G3	518	2	176020986	176066490	323	104	-0.61331	3.70E-01	8.61E-01	6.53E-01	7.30E-01

Using data from 362924 unrelated white British UK Biobank participants.
[a] The number of SNPs annotated to the gene
[b] The number of parameters used in the model
[c] The Z-value for the gene, based on its (permutation) p-value
[d] P-value derived from SNP-wise mean $\chi 2$ model
[e] P-value derived from SNP-wise top $\chi 2$ model
[f] P-value derived from principal components linear regression model
[g] Aggregate p-value derived from all three methods above (*i.e.* d – f)
Gene-based significance threshold ($p<2.87\text{x}10^{-6}$) (24)

Supplemental Table 4: PheWAS (Phenome Wide Association Study) for variant (A) rs111748421, (B) rs12427846, (C) The *SMAD9* gene, using on gene-based test of association

ATLAS.ID	PMID	YEAR	DOMAIN	TRAIT	INCA	DECA	P-value	N
**rs111748421**								
4092	29844566	2018	Cognitive	Reaction time	G	A	6.28E-03	330069
4086	30297969	2018	Endocrine	Type 2 Diabetes (adjusted for BMI)	G	A	2.10E-02	898130
2031	28067908	2017	Gastrointestinal	Inflammatory Bowel Disease	G	A	2.58E-03	59957
2030	28067908	2017	Gastrointestinal	Ulcerative colitis	G	A	3.16E-03	45975
67	26192919	2015	Gastrointestinal	Inflammatory Bowel Disease	G	A	2.24E-02	34652
69	26192919	2015	Gastrointestinal	Ulcerative Colitis	G	A	3.73E-02	27432
3834	27863252	2016	Immunological	Basophil percentage of white cells (two-way meta)	A	G	4.88E-02	131863
4082	30239722	2018	Metabolic	Waist-hip ratio (adjusted for BMI, female)	G	A	2.23E-02	379501
866	27005778	2016	Metabolic	Fatty acid length	A	G	2.37E-02	13476
864	27005778	2016	Metabolic	22:6, docosahexaenoic acid (DHA)	A	G	3.13E-02	13499
4079	30239722	2018	Metabolic	Waist-hip ratio (female)	G	A	4.39E-02	381152
4070	BioRxiv: https://doi.org/10.1101/261081	2018	Psychiatric	Ever smoker	G	A	3.92E-02	518633
**ATLAS.ID**	**PMID**	**YEAR**	**DOMAIN**	**TRAIT**	**INCA**	**DECA**	**P-value**	**N**
**rs12427846**								
3225	Watanabe et al	2017	Activities	Hands-free device/speakerphone use with mobile phone in last 3 month	T	C	4.98E-04	324453
3585	Watanabe et al	2017	Activities	Types of physical activity in last 4 weeks: Walking for pleasure (not as a means of transport)	T	C	8.84E-04	384450
3213	Watanabe et al	2017	Activities	Duration walking for pleasure	C	T	5.52E-03	275181
3556	Watanabe et al	2017	Activities	Medication for cholesterol, blood pressure, diabetes, or take exogenous hormones: Hormone replacement therapy	T	C	1.23E-02	207533
3338	Watanabe et al	2017	Activities	Years since last cervical smear test (female)	C	T	2.20E-02	182552
3609	Watanabe et al	2017	Activities	Treatment/medication code: omega-3/fish oil supplement	T	C	2.99E-02	280443
4068	BioRxiv: https://doi.org/10.1101/261081	2018	Activities	Automobile speeding propensity	T	C	3.00E-02	404291
3613	Watanabe et al	2017	Activities	Treatment/medication code: simvastatin	C	T	3.48E-02	280443
4072	BioRxiv: https://doi.org/10.1101/261081	2018	Activities	First PC of the four riskly behaviours	T	C	4.13E-02	315894
3563	Watanabe et al	2017	Activities	Vitamin and mineral supplements: Vitamin C	C	T	4.30E-02	384452
3542	Watanabe et al	2017	Activities	Reason for glasses/contact lenses: For 'astigmatism'	C	T	4.81E-02	78647
3330	Watanabe et al	2017	Body Structures	Fractured/broken bones in last 5 years	T	C	4.23E-02	384446

3925	28714975	2017	Cardiovascular	Coronary artery disease (SOFT definition including angina)	C	T	8.79E-04	148815
3550	Watanabe et al	2017	Cardiovascular	Vascular/heart problems diagnosed by doctor: Angina	C	T	2.55E-03	385699
3598	Watanabe et al	2017	Cardiovascular	Non-cancer illness code, self-reported: angina	C	T	8.47E-03	289307
3693	Watanabe et al	2017	Cardiovascular	Diagnoses - secondary ICD10: I25 Chronic ischemic heart disease	C	T	2.50E-02	244890
4067	30038396	2018	Cognitive	Cognitive performance	T	C	3.89E-02	257828
3496	Watanabe et al	2017	Dermatological	Hair colour (natural, before greying): Red	T	C	1.28E-02	385603
3632	Watanabe et al	2017	Environment	Illnesses of father: Chronic bronchitis/emphysema	C	T	7.75E-03	355137
3639	Watanabe et al	2017	Environment	Illnesses of mother: Stroke	C	T	1.01E-02	367939
3195	Watanabe et al	2017	Environment	Average total household income before tax	T	C	1.04E-02	332594
3634	Watanabe et al	2017	Environment	Illnesses of father: Diabetes	T	C	1.12E-02	355137
3522	Watanabe et al	2017	Environment	Current employment status: Unable to work because of sickness or disability	C	T	4.60E-02	385138
3350	Watanabe et al	2017	Environment	Bilateral oophorectomy (both ovaries removed) (female)	C	T	4.75E-02	205791
2032	28067912	2017	Gastrointestinal	Prognosis in Crohn's Disease	C	T	3.14E-02	2734
3699	Watanabe et al	2017	Gastrointestinal	Diagnoses - secondary ICD10: K57 Diverticular disease of intestine	C	T	3.59E-02	244890
3670	Watanabe et al	2017	Gastrointestinal	Diagnoses - main ICD10: K29 Gastritis and duodenitis	C	T	4.56E-02	300791
3856	27863252	2016	Immunological	Myeloid white cell count (two-way meta)	C	T	7.49E-03	130268
3841	27863252	2016	Immunological	Granulocyte count (two-way meta)	C	T	7.88E-03	130875
3904	27863252	2016	Immunological	White blood cell count (three-way meta)	C	T	8.25E-03	172435
3857	27863252	2016	Immunological	Sum neutrophil eosinophil count (two-way meta)	C	T	1.11E-02	131409
3836	27863252	2016	Immunological	Sum basophil neutrophil count (two-way meta)	C	T	1.22E-02	131031
3868	27863252	2016	Immunological	White blood cell count (two-way meta)	C	T	1.34E-02	131969
998	27989323	2017	Immunological	Monocyte chemotactic protein-1 (CCL2)	C	T	1.37E-02	8293
3859	27863252	2016	Immunological	Neutrophil count (two-way meta)	C	T	1.42E-02	131564
3892	27863252	2016	Immunological	Myeloid white cell count (three-way meta)	C	T	1.69E-02	169219
3877	27863252	2016	Immunological	Granulocyte count (three-way meta)	C	T	1.82E-02	169822
3893	27863252	2016	Immunological	Sum neutrophil eosinophil count (three-way meta)	C	T	2.41E-02	170384
3872	27863252	2016	Immunological	Sum basophil neutrophil count (three-way meta)	C	T	2.62E-02	170143
3895	27863252	2016	Immunological	Neutrophil count (three-way meta)	C	T	2.86E-02	170702
3897	27863252	2016	Immunological	Plateletcrit (three-way meta)	C	T	3.10E-02	164339
4135	29403010	2018	Immunological	Red blood cell count	T	C	3.39E-02	108794
4132	29403010	2018	Immunological	Basophil count	T	C	4.89E-02	62076
3470	Watanabe et al	2017	Metabolic	Impedance measures - Trunk fat-free mass	T	C	3.82E-05	379507
3471	Watanabe et al	2017	Metabolic	Impedance measures - Trunk predicted mass	T	C	4.02E-05	379469
3444	Watanabe et al	2017	Metabolic	Impedance measures - Whole body water mass	T	C	7.31E-05	379835
3443	Watanabe et al	2017	Metabolic	Impedance measures - Whole body fat-free mass	T	C	8.26E-05	379804
3446	Watanabe et al	2017	Metabolic	Impedance measures - Basal metabolic rate	T	C	2.24E-04	379821

3467	Watanabe et al	2017	Metabolic	Impedance measures - Arm predicted mass (left)	T	C	2.39E-04	379638
3466	Watanabe et al	2017	Metabolic	Impedance measures - Arm fat-free mass (left)	T	C	2.40E-04	379653
3459	Watanabe et al	2017	Metabolic	Impedance measures - Leg predicted mass (left)	T	C	4.53E-04	379761
3458	Watanabe et al	2017	Metabolic	Impedance measures - Leg fat-free mass (left)	T	C	4.62E-04	379766
3463	Watanabe et al	2017	Metabolic	Impedance measures - Arm predicted mass (right)	T	C	8.11E-04	379716
3462	Watanabe et al	2017	Metabolic	Impedance measures - Arm fat-free mass (right)	T	C	8.14E-04	379723
3455	Watanabe et al	2017	Metabolic	Impedance measures - Leg predicted mass (right)	T	C	8.44E-04	379793
3454	Watanabe et al	2017	Metabolic	Impedance measures - Leg fat-free mass (right)	T	C	9.71E-04	379793
3440	Watanabe et al	2017	Metabolic	Impedance measures - Weight	T	C	5.13E-03	379840
932	27005778	2016	Metabolic	Pyruvate	C	T	6.36E-03	24756
906	27005778	2016	Metabolic	Lactate	C	T	6.86E-03	24871
878	27005778	2016	Metabolic	Histidine	C	T	1.03E-02	19244
3436	Watanabe et al	2017	Metabolic	Weight	T	C	1.11E-02	385473
3186	Watanabe et al	2017	Metabolic	Hip circumference	T	C	1.74E-02	385887
4112	29403010	2018	Metabolic	Sodium	C	T	2.33E-02	127304
3449	Watanabe et al	2017	Metabolic	Impedance measures - Impedance of leg (left)	C	T	2.54E-02	379807
3447	Watanabe et al	2017	Metabolic	Impedance measures - Impedance of whole body	C	T	3.13E-02	379792
873	27005778	2016	Metabolic	Glycerol	C	T	3.91E-02	20235
3190	Watanabe et al	2017	Mortality	Number of self-reported non-cancer illnesses	C	T	3.77E-03	386581
3707	Watanabe et al	2017	Mortality	Diagnoses - secondary ICD10: Z72 Problems related to lifestyle	C	T	2.31E-02	244890
3713	Watanabe et al	2017	Mortality	Diagnoses - secondary ICD10: Z88 Allergy status to drug/meds/biol subst	T	C	4.13E-02	244890
3666	Watanabe et al	2017	Neoplasms	Diagnoses - main ICD10: D12 Benign neoplasm of colon, rectum, anus	C	T	8.20E-04	300791
1222	25607358	2015	Neurological	Mean Hippocampus	T	C	2.36E-03	13171
3831	27455348	2016	Neurological	Amyotrophic lateral sclerosis (linear mixed model)	C	T	6.75E-03	36052
4010	29566793	2018	Neurological	Amyotrophic lateral sclerosis	C	T	6.81E-03	80610
3830	27455348	2016	Neurological	Amyotrophic lateral sclerosis (meta-analysis)	C	T	7.77E-03	36052
3953	28931804	2017	Neurological	Amyotrophic lateral sclerosis (LMM)	C	T	1.79E-02	40136
3952	28931804	2017	Neurological	Amyotrophic lateral sclerosis (logistic)	C	T	2.32E-02	40136
1219	25607358	2015	Neurological	Mean Accumbens	T	C	2.82E-02	13171
1223	25607358	2015	Neurological	Mean Pallidum	T	C	4.20E-02	13171
1220	25607358	2015	Neurological	Mean Amygdala	T	C	4.32E-02	13171
3240	Watanabe et al	2017	Nutritional	Cooked vegetable intake	T	C	2.18E-02	374858
3250	Watanabe et al	2017	Nutritional	Pork intake	C	T	3.56E-02	384328
3254	Watanabe et al	2017	Nutritional	Salt added to food	T	C	4.69E-02	386322
4015	29760442	2018	Ophthalmological	Central corneal thickness	C	T	2.44E-02	17803
3263	Watanabe et al	2017	Psychiatric	Average weekly champagne plus white wine intake	T	C	6.98E-03	273869

3239	Watanabe et al	2017	Psychiatric	Exposure to tobacco smoke outside home	C	T	1.06E-02	327794
4042	28761083	2018	Psychiatric	Obsessive compulsive disorder	C	T	1.23E-02	9725
3393	Watanabe et al	2017	Psychiatric	Number of depression episodes	C	T	1.35E-02	49205
3408	Watanabe et al	2017	Psychiatric	Average weekly intake of other alcoholic drinks	C	T	2.14E-02	91288
3725	Watanabe et al	2017	Psychiatric	Frequency of drinking alcohol	T	C	2.35E-02	126656
3416	Watanabe et al	2017	Psychiatric	Bipolar and major depression status	C	T	2.36E-02	93296
1141	27494321	2016	Psychiatric	Chronotype	C	T	3.10E-02	128266
1174	26955885	2016	Psychiatric	Extreme chronotype	C	T	3.70E-02	100420
3722	Watanabe et al	2017	Psychiatric	Frequency of feeling guilt or remorse after drinking alcohol in last year	T	C	4.22E-02	69203
4069	BioRxiv: https://doi.org/10.1101/261081	2018	Psychiatric	Drinks per week	T	C	4.88E-02	414343
3343	Watanabe et al	2017	Reproduction	Age at first live birth (female)	T	C	7.83E-04	141051
3344	Watanabe et al	2017	Reproduction	Age at last live birth (female)	T	C	4.51E-03	140855
3320	Watanabe et al	2017	Respiratory	Chest pain or discomfort	C	T	1.32E-03	382525
3319	Watanabe et al	2017	Respiratory	Wheeze or whistling in the chest in last year	C	T	3.41E-03	379150
3372	Watanabe et al	2017	Respiratory	Age hay fever, rhinitis or eczema diagnosed	T	C	5.92E-03	77452
3599	Watanabe et al	2017	Respiratory	Non-cancer illness code, self-reported: asthma	C	T	9.40E-03	289307
3552	Watanabe et al	2017	Respiratory	Blood clot, DVT, bronchitis, emphysema, asthma, rhinitis, eczema, allergy diagnosed by doctor: Asthma	C	T	1.16E-02	385822
3397	Watanabe et al	2017	Respiratory	Shortness of breath walking on level ground	C	T	2.73E-02	126962
3979	28869591	2017	Skeletal	Estimated BMD	C	T	**3.60E-10**	142487
3971	29304378	2018	Skeletal	Total body BMD	C	T	**2.35E-08**	66628
3974	29304378	2018	Skeletal	Total body BMD (30-45 years old)	C	T	1.85E-05	10062
3187	Watanabe et al	2017	Skeletal	Standing height	T	C	5.84E-05	385748
84	26367794	2015	Skeletal	Forearm BMD	C	T	3.79E-04	32965
3271	Watanabe et al	2017	Skeletal	Comparative height size at age 10	T	C	7.35E-04	380167
3976	29304378	2018	Skeletal	Total body BMD (15 or younger)	C	T	2.69E-03	11807
3412	Watanabe et al	2017	Skeletal	Sitting height	T	C	9.19E-03	385393
3972	29304378	2018	Skeletal	Total body BMD (60 or older)	C	T	2.25E-02	22504
3975	29304378	2018	Skeletal	Total body BMD (15-30 years old)	C	T	2.36E-02	4180
86	26367794	2015	Skeletal	Lumbar Spine BMD	C	T	2.40E-02	32965
3973	29304378	2018	Skeletal	Total body BMD (45-60 years old)	C	T	4.03E-02	18805
3517	Watanabe et al	2017	Social Interactions	How are people in household related to participant: Husband, wife or partner	T	C	4.71E-03	312871
3358	Watanabe et al	2017	Social Interactions	Father's age	T	C	2.88E-02	86941

ATLAS.ID	PMID	YEAR	DOMAIN	TRAIT	P-value	N
***SMAD9* gene**						
3215	Watanabe et al	2017	Activities	Duration of light DIY	2.30E-03	190862
3225	Watanabe et al	2017	Activities	Hands-free device/speakerphone use with mobile phone in last 3 month	8.48E-03	324453
3213	Watanabe et al	2017	Activities	Duration walking for pleasure	9.14E-03	275181
3622	Watanabe et al	2017	Activities	Treatment/medication code: cod liver oil capsule	2.14E-02	280443
3585	Watanabe et al	2017	Activities	Types of physical activity in last 4 weeks: Walking for pleasure (not as a means of transport)	2.61E-02	384450
1982	17903295	2007	Aging	Walking speed (at exam 7, FBAT)	3.63E-02	764
4050	28358823	2017	Body Structures	Anterior cruciate ligament rupture (fixed effect model)	5.40E-03	99342
1577	17903303	2007	Cardiovascular	Maximum coronary artery calcification - Agatston score (at exam 7, GEE, adjusted for age and sex)	3.96E-03	680
1575	17903303	2007	Cardiovascular	Mean coronary artery calcification - Agatston score (at exam 7, GEE, adjusted for age and sex)	5.56E-03	680
1576	17903303	2007	Cardiovascular	Maximum coronary artery calcification - Agatston score (at exam 7, FBAT, adjusted for age and sex)	6.07E-03	680
3925	28714975	2017	Cardiovascular	Coronary artery disease (SOFT definition including angina)	6.22E-03	148815
1574	17903303	2007	Cardiovascular	Mean coronary artery calcification - Agatston score (at exam 7, FBAT, adjusted for age and sex)	8.40E-03	680
3986	29497042	2018	Cardiovascular	Heart rate recovery at 20 secnds	3.18E-02	58818
3693	Watanabe et al	2017	Cardiovascular	Diagnoses - secondary ICD10: I25 Chronic ischemic heart disease	3.89E-02	244890
1578	17903303	2007	Cardiovascular	Maximum coronary artery calcification - Agatston score (at exam 7, FBAT, adjusted for multivariable)	4.93E-02	678
2416	28240269	2017	Cell	FOLH1 - Glutamate carboxypeptidase 2	2.74E-04	1000
2262	28240269	2017	Cell	CTF1 - Cardiotrophin-1	6.78E-04	1000
3058	28240269	2017	Cell	IL22RA2 - Interleukin-22 receptor subunit alpha-2	5.46E-03	1000
3156	28240269	2017	Cell	TNFSF14 - Tumor necrosis factor ligand superfamily member 14	6.39E-03	1000
2309	28240269	2017	Cell	IL17RA - Interleukin-17 receptor A	6.67E-03	1000
2598	28240269	2017	Cell	HGFAC - Hepatocyte growth factor activator	7.64E-03	1000
2301	28240269	2017	Cell	CXCL5 - C-X-C motif chemokine 5	9.13E-03	1000
2757	28240269	2017	Cell	TNC - Tenascin	1.33E-02	1000
2444	28240269	2017	Cell	GFRA1 - GDNF family receptor alpha-1	1.33E-02	1000
2732	28240269	2017	Cell	CCL24 - C-C motif chemokine 24	1.49E-02	1000
2196	28240269	2017	Cell	GDF11 - Growth/differentiation factor 11	1.66E-02	1000
2195	28240269	2017	Cell	FGF9 - Fibroblast growth factor 9	1.68E-02	1000
2489	28240269	2017	Cell	PAK3 - Serine/threonine-protein kinase PAK 3	1.80E-02	1000
2271	28240269	2017	Cell	SERPINE1 - Plasminogen activator inhibitor 1	1.80E-02	1000
2398	28240269	2017	Cell	GNLY - Granulysin	2.03E-02	1000
2411	28240269	2017	Cell	ASAH2 - Neutral ceramidase	2.05E-02	1000
2391	28240269	2017	Cell	C2 - Complement C2	2.12E-02	1000
2325	28240269	2017	Cell	CAST - Calpastatin	2.15E-02	1000
2901	28240269	2017	Cell	IL6 - Interleukin-6	2.19E-02	1000

3167	28240269	2017	Cell	CDH15 - Cadherin-15	2.30E-02	1000
3158	28240269	2017	Cell	NLGN4X - Neuroligin-4, X-linked	2.73E-02	1000
2127	28240269	2017	Cell	IL1RAP - Interleukin-1 Receptor accessory protein	2.85E-02	1000
2311	28240269	2017	Cell	IL1RL2 - Interleukin-1 receptor-like 2	3.28E-02	1000
2329	28240269	2017	Cell	FTH1 FTL - Ferritin	3.37E-02	1000
2988	28240269	2017	Cell	DPT - Dermatopontin	3.37E-02	1000
2502	28240269	2017	Cell	TPSG1 - Tryptase gamma	3.49E-02	1000
3097	28240269	2017	Cell	HAVCR2 - Hepatitis A virus cellular receptor 2	3.75E-02	1000
2230	28240269	2017	Cell	KLK4 - Kallikrein-4	3.96E-02	1000
2216	28240269	2017	Cell	ACE2 - Angiotensin-converting enzyme 2	4.03E-02	1000
3070	28240269	2017	Cell	LAG3 - Lymphocyte activation gene 3 protein	4.17E-02	1000
3164	28240269	2017	Cell	BRF1 - Transcription factor IIIB 90 kDa subunit	4.21E-02	1000
2978	28240269	2017	Cell	ERAP1 - Endoplasmic reticulum aminopeptidase 1	4.34E-02	1000
2455	28240269	2017	Cell	NTN4 - Netrin-4	4.56E-02	1000
2982	28240269	2017	Cell	CA1 - Carbonic anhydrase 1	4.64E-02	1000
2071	28240269	2017	Cell	APOE - Apolipoprotein E	4.74E-02	1000
2742	28240269	2017	Cell	IL20 - Interleukin-20	4.89E-02	1000
2041	27656889	2016	Cognitive	Attention function - Hit reaction time - Child	4.21E-02	1655
3498	Watanabe et al	2017	Dermatological	Hair colour (natural, before greying): Dark brown	1.21E-04	385603
3499	Watanabe et al	2017	Dermatological	Hair colour (natural, before greying): Black	1.31E-03	385603
3274	Watanabe et al	2017	Dermatological	Ease of skin tanning	1.87E-03	378364
3497	Watanabe et al	2017	Dermatological	Hair colour (natural, before greying): Light brown	2.44E-03	385603
3273	Watanabe et al	2017	Dermatological	Skin colour	5.31E-03	381433
3496	Watanabe et al	2017	Dermatological	Hair colour (natural, before greying): Red	9.91E-03	385603
1698	17903292	2007	Endocrine	Dehydroepiandrosterone sulfate (at exam 3, FBAT, adjusted for age and sex)	2.70E-02	850
1709	17903292	2007	Endocrine	Luteinizing hormone (at exam 3, GEE, adjusted for multivariable)	3.45E-02	508
3639	Watanabe et al	2017	Environment	Illnesses of mother: Stroke	8.34E-03	367939
3522	Watanabe et al	2017	Environment	Current employment status: Unable to work because of sickness or disability	2.05E-02	385138
3195	Watanabe et al	2017	Environment	Average total household income before tax	3.07E-02	332594
3632	Watanabe et al	2017	Environment	Illnesses of father: Chronic bronchitis/emphysema	3.72E-02	355137
2004	20639880	2010	Gastrointestinal	Primary biliary cirrhosis	3.73E-02	1551
2047	26962152	2016	Gastrointestinal	Periodontal complex trait 2	3.92E-02	975
1610	17903294	2007	Hematological	von Willebrand Factor (at exam 5, FBAT, adjusted for age and sex)	1.73E-02	883
1612	17903294	2007	Hematological	von Willebrand Factor (at exam 5, FBAT, adjusted for multivariable)	3.77E-02	883
1013	27989323	2017	Immunological	Vascular endothelial growth factor	3.16E-04	7118
1650	17903293	2007	Immunological	Tumor necrosis factor alpha (at exam 7, FBAT, adjusted for age and sex)	1.88E-03	753

1652	17903293	2007	Immunological	Tumor necrosis factor alpha (at exam 7, FBAT, adjusted for multivariable)	2.00E-03	753
1651	17903293	2007	Immunological	Tumor necrosis factor alpha (at exam 7, GEE, adjusted for age and sex)	6.63E-03	753
310	25772697	2015	Immunological	CD4nv:preTh17	6.94E-03	669
998	27989323	2017	Immunological	Monocyte chemotactic protein-1 (CCL2)	9.59E-03	8293
981	27989323	2017	Immunological	Interleukin-10	1.20E-02	7681
1653	17903293	2007	Immunological	Tumor necrosis factor alpha (at exam 7, GEE, adjusted for multivariable)	1.29E-02	753
983	27989323	2017	Immunological	Interleukin-13	1.35E-02	3557
3802	19853236	2009	Immunological	Mean erythrocyte cell volume	1.35E-02	5945
1003	27989323	2017	Immunological	Macrophage inflammatory protein (CCL3)	1.51E-02	3522
3841	27863252	2016	Immunological	Granulocyte count (two-way meta)	1.82E-02	130875
3836	27863252	2016	Immunological	Sum basophil neutrophil count (two-way meta)	2.25E-02	131031
3857	27863252	2016	Immunological	Sum neutrophil eosinophil count (two-way meta)	2.47E-02	131409
3859	27863252	2016	Immunological	Neutrophil count (two-way meta)	2.71E-02	131564
3856	27863252	2016	Immunological	Myeloid white cell count (two-way meta)	2.74E-02	130268
1625	17903293	2007	Immunological	Intercellular adhesion molecule-1 (at exam 7, GEE, adjusted for multivariable)	3.19E-02	1006
3868	27863252	2016	Immunological	White blood cell count (two-way meta)	3.30E-02	131969
344	25772697	2015	Immunological	NKearly:335+314-	3.72E-02	669
982	27989323	2017	Immunological	Interleukin-12p70	4.12E-02	8270
1623	17903293	2007	Immunological	Intercellular adhesion molecule-1 (at exam 7, GEE, adjusted for age and sex)	4.30E-02	1006
266	25772697	2015	Immunological	DPT:Exhausted	4.84E-02	669
3470	Watanabe et al	2017	Metabolic	Impedance measures - Trunk fat-free mass	8.46E-05	379507
3471	Watanabe et al	2017	Metabolic	Impedance measures - Trunk predicted mass	9.53E-05	379469
3443	Watanabe et al	2017	Metabolic	Impedance measures - Whole body fat-free mass	1.92E-04	379804
3444	Watanabe et al	2017	Metabolic	Impedance measures - Whole body water mass	2.30E-04	379835
465	24816252	2014	Metabolic	Lipid::Bile acid metabolism::deoxycholate	2.94E-04	5194
3466	Watanabe et al	2017	Metabolic	Impedance measures - Arm fat-free mass (left)	6.58E-04	379653
3467	Watanabe et al	2017	Metabolic	Impedance measures - Arm predicted mass (left)	7.45E-04	379638
3446	Watanabe et al	2017	Metabolic	Impedance measures - Basal metabolic rate	1.05E-03	379821
4126	29403010	2018	Metabolic	Creatine kinase	1.10E-03	106080
403	24816252	2014	Metabolic	Amino acid::Tryptophan metabolism::C-glycosyltryptophan*	1.53E-03	7786
3459	Watanabe et al	2017	Metabolic	Impedance measures - Leg predicted mass (left)	2.28E-03	379761
3463	Watanabe et al	2017	Metabolic	Impedance measures - Arm predicted mass (right)	2.35E-03	379716
3462	Watanabe et al	2017	Metabolic	Impedance measures - Arm fat-free mass (right)	2.59E-03	379723
3458	Watanabe et al	2017	Metabolic	Impedance measures - Leg fat-free mass (left)	2.65E-03	379766
1680	17903293	2007	Metabolic	Atrial natriuretic peptide (at exam 6, FBAT, adjusted for multivariable)	3.17E-03	938
3455	Watanabe et al	2017	Metabolic	Impedance measures - Leg predicted mass (right)	3.77E-03	379793

1445	17903299	2007	Metabolic	Low-density lipoprotein cholesterol (at exam 2, GEE, adjusted for age and sex)	3.93E-03	1056
615	24816252	2014	Metabolic	Xenobiotics::Benzoate metabolism::4-vinylphenol sulfate	4.08E-03	7483
1443	17903299	2007	Metabolic	Low-density lipoprotein cholesterol (at exam 1, GEE, adjusted for age and sex)	4.32E-03	1056
3454	Watanabe et al	2017	Metabolic	Impedance measures - Leg fat-free mass (right)	4.34E-03	379793
494	24816252	2014	Metabolic	Lipid::Fatty acid metabolism (also BCAA metabolism)::butyrylcarnitine	5.45E-03	7796
1441	17903299	2007	Metabolic	Low-density lipoprotein cholesterol (at exam 1, GEE, adjusted for multivariable)	8.23E-03	1056
589	24816252	2014	Metabolic	Nucleotide::Pyrimidine metabolism, uracil containing::pseudouridine	8.97E-03	7785
4119	29403010	2018	Metabolic	Aspartate aminotransferase	1.01E-02	134154
1392	17903299	2007	Metabolic	High-density lipoprotein 2 cholesterol (at exam 4, FBAT, adjusted for multivariable)	1.04E-02	955
932	27005778	2016	Metabolic	Pyruvate	1.07E-02	24756
625	24816252	2014	Metabolic	Xenobiotics::Drug::ibuprofen	1.12E-02	1976
1447	17903299	2007	Metabolic	Low-density lipoprotein cholesterol (at exam 3, GEE, adjusted for age and sex)	1.26E-02	1056
1433	17903299	2007	Metabolic	High-density lipoprotein particle size by NMR (at exam 4, GEE, adjusted for multivariable)	1.27E-02	851
729	24816252	2014	Metabolic	Cofactors and vitamins::Hemoglobin and porphyrin metabolism::X-11793--oxidized bilirubin*	1.32E-02	7611
1359	17903299	2007	Metabolic	Total cholesterol (at exam 1, GEE, adjusted for age and sex)	1.39E-02	1069
377	24816252	2014	Metabolic	Amino acid::Glutathione metabolism::5-oxoproline	1.53E-02	7802
641	24816252	2014	Metabolic	Xenobiotics::Xanthine metabolism::1,7-dimethylurate	1.58E-02	6057
436	24816252	2014	Metabolic	Carbohydrate::Fructose, mannose, galactose, starch, and sucrose metabolism::fructose	1.72E-02	7781
406	24816252	2014	Metabolic	Amino acid::Tryptophan metabolism::indolepropionate	1.77E-02	7803
4123	29403010	2018	Metabolic	Activated partial thromboplastin time	1.94E-02	37767
1375	17903299	2007	Metabolic	Total cholesterol / High-density lipoprotein cholesterol ratio (exam 1, GEE, adjusted for age and sex)	2.01E-02	1060
1379	17903299	2007	Metabolic	Total cholesterol / High-density lipoprotein cholesterol ratio (exam 3, GEE, adjusted for age and sex)	2.06E-02	1060
1684	17903293	2007	Metabolic	Brain natriuretic peptide (at exam 6, FBAT, adjusted for multivariable)	2.06E-02	938
1357	17903299	2007	Metabolic	Total cholesterol (at exam 1, GEE, multivariate adjusted)	2.16E-02	1069
1678	17903293	2007	Metabolic	Atrial natriuretic peptide (at exam 6, FBAT, adjusted for age and sex)	2.25E-02	938
1361	17903299	2007	Metabolic	Total cholesterol (at exam 2, GEE, adjusted for age and sex)	2.29E-02	1069
1490	17903299	2007	Metabolic	Plasma Apolipoprotein E level (at exam 5, FBAT, adjusted for age and sex)	2.31E-02	744
398	24816252	2014	Metabolic	Amino acid::Phenylalanine & tyrosine metabolism::phenylacetylglutamine	2.46E-02	7812
1393	17903299	2007	Metabolic	High-density lipoprotein 2 cholesterol (at exam 4, GEE, adjusted for multivariable)	2.57E-02	955
642	24816252	2014	Metabolic	Xenobiotics::Xanthine metabolism::1-methylurate	2.82E-02	5520
1363	17903299	2007	Metabolic	Total cholesterol (at exam 3, GEE, adjusted for age and sex)	2.96E-02	1069
639	24816252	2014	Metabolic	Xenobiotics::Tobacco metabolite::cotinine	3.31E-02	1270
554	24816252	2014	Metabolic	Lipid::Medium chain fatty acid::5-dodecenoate (12:1n7)	3.37E-02	7770
522	24816252	2014	Metabolic	Lipid::Long chain fatty acid::myristoleate (14:1n5)	3.48E-02	7804
1435	17903299	2007	Metabolic	High-density lipoprotein particle size by NMR (at exam 4, GEE, adjusted for age and sex)	3.68E-02	851
1488	17903299	2007	Metabolic	Plasma Apolipoprotein E level (at exam 5, FBAT, adjusted for multivariable)	3.74E-02	744

1373	17903299	2007	Metabolic	Total cholesterol / High-density lipoprotein cholesterol ratio (at exam 1, GEE, adjusted for multivariable)	3.75E-02	1060
906	27005778	2016	Metabolic	Lactate	3.82E-02	24871
1345	17903299	2007	Metabolic	Plasma Apolipoprotein A-I level (GEE, multivariate adjusted)	3.84E-02	997
1449	17903299	2007	Metabolic	Low-density lipoprotein cholesterol (at exam 4, GEE, adjusted for age and sex)	4.01E-02	1056
1432	17903299	2007	Metabolic	High-density lipoprotein particle size by NMR (at exam 4, FBAT, adjusted for multivariable)	4.02E-02	851
1395	17903299	2007	Metabolic	High-density lipoprotein 2 cholesterol (at exam 4, GEE, adjusted for age and sex)	4.03E-02	955
1425	17903299	2007	Metabolic	Large high-density lipoprotein by NMR (at exam 4, GEE, adjusted for multivariable)	4.21E-02	851
561	24816252	2014	Metabolic	Lipid::Medium chain fatty acid::undecanoate (11:0)	4.51E-02	7500
191	26831199	2016	Metabolic	Estimated glomerular filtration rate based on serum creatinine	4.69E-02	113814
196	26831199	2016	Metabolic	Estimated glomerular filtration rate based on serum creatinine	4.69E-02	16474
4112	29403010	2018	Metabolic	Sodium	4.81E-02	127304
1481	17903299	2007	Metabolic	Mean low-density lipoprotein cholesterol from exam 1-7 (GEE, adjusted for multivariable)	4.96E-02	1086
619	24816252	2014	Metabolic	Xenobiotics::Chemical::glycerol 2-phosphate	4.99E-02	5912
1207	24688116	2014	Mortality	Longevity (>= 85 yrs vs < 65 yrs)	2.42E-02	20518
4097	29471430	2018	Neoplasms	Colorectal cancer	5.30E-04	33870
3666	Watanabe et al	2017	Neoplasms	Diagnoses - main ICD10: D12 Benign neoplasm of colon, rectum, anus and anal canal	6.61E-03	300791
3831	27455348	2016	Neurological	Amyotrophic lateral sclerosis (linear mixed model)	2.51E-03	36052
3830	27455348	2016	Neurological	Amyotrophic lateral sclerosis (meta-analysis)	3.73E-03	36052
3952	28931804	2017	Neurological	Amyotrophic lateral sclerosis (logistic)	4.80E-03	40136
3953	28931804	2017	Neurological	Amyotrophic lateral sclerosis (LMM)	4.85E-03	40136
1222	25607358	2015	Neurological	Mean Hippocampus	1.07E-02	13171
1907	17903297	2007	Neurological	Hippocampal volume (GEE, adjusted for multivariable)	2.21E-02	327
4010	29566793	2018	Neurological	Amyotrophic lateral sclerosis	2.61E-02	80610
1941	17903297	2007	Neurological	Hippocampal volume (GEE, adjusted for multivariable with APOE)	2.61E-02	327
1925	17903297	2007	Neurological	Hippocampal volume (GEE, adjusted for multivariable)	3.20E-02	327
3241	Watanabe et al	2017	Nutritional	Salad / raw vegetable intake	6.30E-06	363780
3251	Watanabe et al	2017	Nutritional	Cheese intake	9.82E-03	377082
3258	Watanabe et al	2017	Nutritional	Water intake	3.64E-02	357000
3240	Watanabe et al	2017	Nutritional	Cooked vegetable intake	4.21E-02	374858
2025	22952603	2012	Psychiatric	10 mg response to amphetamine	1.87E-03	381
3239	Watanabe et al	2017	Psychiatric	Exposure to tobacco smoke outside home	7.36E-03	327794
4042	28761083	2018	Psychiatric	Obsessive compulsive disorder	1.22E-02	9725
3779	Watanabe et al	2017	Psychiatric	Traumatic events - Witnessed sudden violent death	1.36E-02	126595
3263	Watanabe et al	2017	Psychiatric	Average weekly champagne plus white wine intake	1.73E-02	273869
3439	Watanabe et al	2017	Psychiatric	Tobacco smoking	2.41E-02	97172

3509	Watanabe et al	2017	Psychiatric	Reason for reducing amount of alcohol drunk: Illness or ill health	2.72E-02	142645
4011	29662059	2018	Psychiatric	Broad depression	2.84E-02	322580
2024	23089632	2013	Psychiatric	Alcohol dependence	3.86E-02	2322
3656	Watanabe et al	2017	Psychiatric	Alcohol - Alcohol drinker status: Never	4.02E-02	386082
3301	Watanabe et al	2017	Psychiatric	Seen doctor (GP) for nerves, anxiety, tension or depression	4.20E-02	383771
3234	Watanabe et al	2017	Psychiatric	Daytime dozing / sleeping (narcolepsy)	4.22E-02	384879
2026	22952603	2012	Psychiatric	Baseline positive affect factor score	4.23E-02	381
20	20418890	2010	Psychiatric	Number of cigarettes smoked per day	4.43E-02	38181
2016	24369049	2014	Psychiatric	Lithium response in Bipolar I patients - Alda Scale of 5 to 6	4.55E-02	294
3776	Watanabe et al	2017	Psychiatric	Traumatic events - Been in serious accident believed to be life-threatening	4.68E-02	126665
1275	17903308	2007	Psychiatric	Epworth Sleepiness Scale (GEE, adjusted for age, sex, BMI, usual sleep duration and additional self-reported sleeping disorder)	4.95E-02	721
2028	25390077	2015	Reproduction	Cryptorchidism (group 2)	3.11E-03	1502
3343	Watanabe et al	2017	Reproduction	Age at first live birth (female)	1.33E-02	141051
3344	Watanabe et al	2017	Reproduction	Age at last live birth (female)	1.53E-02	140855
2007	22377632	2012	Reproduction	Parental transmission distortion	2.02E-02	4728
3965	28990592	2017	Reproduction	Gestational weight gain (offspring, total)	2.61E-02	8625
1986	17903295	2007	Reproduction	Age at natural menopause (FBAT)	4.37E-02	438
3320	Watanabe et al	2017	Respiratory	Chest pain or discomfort	1.28E-03	382525
3372	Watanabe et al	2017	Respiratory	Age hay fever, rhinitis or eczema diagnosed	4.47E-03	77452
3979	28869591	2017	Skeletal	Estimated BMD	**2.96E-09**	142487
3971	29304378	2018	Skeletal	Total body BMD	**7.57E-08**	66628
3187	Watanabe et al	2017	Skeletal	Standing height	5.74E-06	385748
4043	30124842	2018	Skeletal	Height	1.07E-05	693529
3974	29304378	2018	Skeletal	Total body BMD (30-45 years old)	8.16E-04	10062
3271	Watanabe et al	2017	Skeletal	Comparative height size at age 10	1.22E-03	380167
3412	Watanabe et al	2017	Skeletal	Sitting height	1.39E-03	385393
84	26367794	2015	Skeletal	Forearm BMD	1.48E-03	32965
3926	28743860	2017	Skeletal	Total-body less head BMD	2.91E-03	10414
1837	17903296	2007	Skeletal	Neck width (GEE, male, adjusted for multivariable)	2.97E-03	477
1850	17903296	2007	Skeletal	Neck average buckling ratio (FBAT, female, adjusted for multivariable)	3.52E-03	618
3976	29304378	2018	Skeletal	Total body BMD (15 or younger)	4.31E-03	11807
1832	17903296	2007	Skeletal	Neck length (FBAT, male, adjusted for multivariable)	6.76E-03	471
1841	17903296	2007	Skeletal	Neck width (GEE, male, adjusted for age)	7.07E-03	477
3928	28743860	2017	Skeletal	Total-body less head BMD and total body lean mass (bivariate meta-analysis)	1.02E-02	10414
1818	17903296	2007	Skeletal	Inter-trochanteric buckling ratio (FBAT, female, adjusted for multivariable)	1.23E-02	596

1894	17903296	2007	Skeletal	Shaft Section Modulus (FBAT, female, adjusted for multivariable)	1.54E-02	599
1826	17903296	2007	Skeletal	Spine Neck BMD (FBAT, female, adjusted for multivariable)	1.56E-02	641
1875	17903296	2007	Skeletal	Shaft average buckling ratio (GEE, female, adjusted for age)	2.26E-02	599
1870	17903296	2007	Skeletal	Shaft average buckling ratio (FBAT, female, adjusted for multivariable)	2.43E-02	599
86	26367794	2015	Skeletal	Lumbar Spine BMD	2.53E-02	32965
1827	17903296	2007	Skeletal	Spine Neck BMD (GEE, female, adjusted for multivariable)	3.66E-02	641
1823	17903296	2007	Skeletal	Inter-trochanteric buckling ratio (GEE, female, adjusted for age)	3.75E-02	596
1854	17903296	2007	Skeletal	Neck average buckling ratio (FBAT, female, adjusted for age)	4.01E-02	618
1025	17903296	2007	Skeletal	Femoral Neck Length (GEE, adjusted for age and sex)	4.37E-02	1090
3358	Watanabe et al	2017	Social Interactions	Father's age	1.50E-02	86941
3577	Watanabe et al	2017	Social Interactions	Social support - Leisure/social activities: Pub or social club	3.65E-02	385280
3517	Watanabe et al	2017	Social Interactions	How are people in household related to participant: Husband, wife or partner	4.19E-02	312871

INCA - is the trait increasing allele. G allele is the Bone Mass increasing allele for rs111748421, C allele is the eBMD increasing allele for rs12427846
Watanabe et al available as pre-print: https://www.biorxiv.org/content/10.1101/500090v1.
P values: Highlighted in bold are associations supported by evidence exceeding p value threshold: (A) rs111748421 $p<5x10^{-8}$, (B) rs12427846 $p<5x10^{-8}$, (C) The *SMAD9* gene $p<2.87x10^{-6}$ (from Morris et al (24)).

Supplemental Table 5: - Murine osteocyte expression by whole transcriptome sequencing of Smad9 and Chrna1 in four bone types (tibia, femur, humerus and calvaria)

MGI Gene symbol	Chrom	Skeletal GO	Tibia		Femur		Humerus		Calvaria		Clean Bone		Bone And Marrow		Expressed in all bone types	Enriched in osteocyte samples	Osteocyte Signature
			Activity	Mean FPKM	Activity	Mean FPKM	Activity	Mean FPKM	Activity	Mean FPKM	Activity	Mean FPKM	Activity	Mean FPKM			
**Smad9**	3	GO:0060348GO:0051216	Active 8/8	6.84	Active 8/8	6.13	Active 8/8	6.96	Active 8/8	2.78	Active 5/5	7.19	Active 1/5	0.37	**Yes**	**Yes**	**Yes**
**Chrna1**	2	GO:0050881	Active 4/8	0.51	Active 2/8	0.44	Inactive	0.25	Inactive	0.05	Inactive	0.22	Inactive	0.05	**No**	**No**	**No**

MGI: Mouse Genome Informatics.
Mouse Ensembl Ids: *Smad9* - ENSMUSG00000027796, *Chrna1*- ENSMUSG00000027107
Skeletal GO: Gene Ontology (http://www.ebi.ac.uk)
Activity: Number of replicates of that bone type with FPKM expression values above active gene threshold.
Clean bone: bone with marrow removed to isolate osteocytes
Bone and marrow: bone cleaned of connective tissue and growth plates with the marrow left intact
Mean FPKM: Mean gene expression in for gene across samples of bone type normalised for gene length and library size (Fragments Per Kilobase per Million mapped reads)

Supplemental Acknowledgements
We would like to thank all study participants who provided DNA and clinical information. Regarding the HBM study, we particularly thank staff at the Wellcome Trust Clinical Research Facility in Birmingham, Royal National Hospital for Rheumatic Diseases in Bath, Cambridge NIHR Biomedical Research Centre and Addenbrooke's Wellcome Trust Clinical Research Facility in Cambridge, Bone Research Unit in Cardiff, Musculoskeletal Research Unit in Bristol, NIHR Bone Biomedical Research Unit in Sheffield and the Brocklehurst Centre for Metabolic Bone Disease in Hull.

Regarding the Anglo-Australasian Genetics Consortium (AOGC) study, we thank the AOGC PIs; Eugene McCloskey (Sheffield, UK), Geoffrey C Nicholson (Geelong, Australia), Richard Eastell (Sheffield, UK), Richard L Prince (Perth, Australia), John A Eisman (Sydney, Australia), Graeme Jones (Hobart, Australia), Philip Sambrook (Sydney, Australia), Ian R Reid (Auckland, New Zealand), Elaine M Dennison (Southampton, UK), John Wark (Geelong, Australia). Furthermore, we thank Barbara Mason and Amanda Horne (Auckland) for patient recruitment; Judith Finigan (Sheffield, UK) for laboratory support and database support; Selina Simpson (Sheffield, UK) for DNA handling; Fatma Gossiel (Sheffield, UK) for DNA handling; Alison Steward and Lana Gibson (Aberdeen, UK) for patient recruitment; Katherine Kolk (Geelong, Australia); Janelle Rampellini (Perth, Australia) for patient recruitment; Jemma Christie (Melbourne, Australia) for patient recruitment; Helen Steane (Hobart, Australia) for patient recruitment; Denia Mang and Ruth Toppler for DNA extraction, DNA handling, and database support (Dubbo/Sydney, Australia); Kate Lowings (Brisbane, Australia) for patient recruitment; and Marieke Brugmans and Leanne Brookes (Brisbane, Australia) for DNA preparation and genotyping. We thank Ms Linda Bradbury (Brisbane, Australia) for support with ethics, governance and recruitment.

The HBM study was supported by the UK NIHR CRN (portfolio number 5163); supporting CLRNs included Birmingham and the Black Country, London South, Norfolk & Suffolk, North and East Yorkshire and Northern Lincolnshire, South Yorkshire, Surrey & Sussex, West Anglia and Western.

The AOGC also received funding from the Australian Cancer Research Foundation and Rebecca Cooper Foundation (Australia). MAB was funded by a National Health and Medical Research Council (Australia) Principal Research Fellowship and ELD was funded by a National Health and Medical Research Council (Australia) Career Development Award (569807). IR is supported by the Health Research Council of New Zealand. The OPUS study was supported by Sanofi-Aventis, Eli Lilly, Novartis, Pfizer, Proctor & Gamble Pharmaceuticals and Roche. The Sydney Twin Study was supported by the National Health and Medical Research Council, Australia. The Dubbo Osteoporosis Epidemiology Study was supported by the Australian National Health and Medical Research Council, MBF Living Well foundation, the Ernst Heine Family Foundation and from untied educational grants from Amgen, Eli Lilly International, GE-Lunar, Merck Australia, Novartis, Sanofi-Aventis Australia and Servier. The Hertfordshire Cohort Study was supported by grants from the Medical Research Council UK & Arthritis Research UK. The Geelong Osteoporosis Study was funded by grants from the Victorian Health Promotion Foundation and the Geelong Region Medical Research Foundation, and the National Health and Medical Research Council, Australia (project grant 628582). The Oxford Osteoporosis Study was funded by Action Research UK.

This research has been conducted using the UK Biobank Resource (accession IDs: 12703). We would like to thank the Wolfson Bioimaging Facility at the University of Bristol, UK, for confocal microscope access and imaging support and Jessica Harris and Sharon Song at the Queensland University of Technology for their help with genotyping.

Supplemental References

1. Gregson CL, Hardcastle SA, Cooper C, Tobias JH. Friend or foe: high bone mineral density on routine bone density scanning, a review of causes and management. Rheumatology. 2013;52(6):968-85.
2. Kellgren J, Lawrence J. Radiological assessment of osteo-arthrosis. Ann Rheum Dis. 1957;16(4):494-502.
3. Gregson CL, Steel S, Yoshida K, Reid DM, Tobias JH. An investigation into the impact of osteoarthritic changes on bone mineral density measurements in patients with High Bone Mass. ASBMR 30th Annual Meeting, Montreal 2008;SA257.
4. Hansen KE, Binkley N, Christian R, Vallarta-Ast N, Krueger D, Drezner MK, et al. Interobserver reproducibility of criteria for vertebral body exclusion. J Bone Miner Res. 2005;20(3):501-8.
5. Little RD, Carulli JP, Del Mastro RG, Dupuis J, Osborne M, Folz C, et al. A mutation in the LDL receptor-related protein 5 gene results in the autosomal dominant high-bone-mass trait. Am J Hum Genet. 2002;70(1):11-9.
6. White J, Yeats A, Skipworth G. Tables for Statisticians: Stanley Thornes, Cheltenham; 1979.
7. Gregson CL, Steel SA, O'Rourke KP, Allan K, Ayuk J, Bhalla A, et al. 'Sink or swim': an evaluation of the clinical characteristics of individuals with high bone mass. Osteo Int. 2012;23(2):643-54.
8. The 59th General Assembly Seoul. World Medical Assembly Declaration of Helsinki. Ethical Principles for Medical Research Involving Human Subjects. Seoul, Korea); 2008.
9. Gregson CL, Paggiosi MA, Crabtree N, Steel SA, McCloskey E, Duncan EL, et al. Analysis of body composition in individuals with high bone mass reveals a marked increase in fat mass in women but not men. J Clin Endocrinol Metab. 2013;98(2):818-28.
10. Gregson CL, Sayers A, Lazar V, Steel S, Dennison EM, Cooper C, et al. The high bone mass phenotype is characterised by a combined cortical and trabecular bone phenotype: Findings from a pQCT case–control study. Bone. 2013;52(1):380-8.
11. Ward KA, Adams JE, Hangartner TN. Recommendations for thresholds for cortical bone geometry and density measurement by peripheral quantitative computed tomography. Calcif Tissue Int. 2005;77(5):275-80.
12. Stratec Medizintechnik GmbH. XCT 2000 Research Manual Software Version 6.20. 2012.
13. Gluer CC, Eastell R, Reid DM, Felsenberg D, Roux C, Barkmann R, et al. Association of five quantitative ultrasound devices and bone densitometry with osteoporotic vertebral fractures in a population-based sample: the OPUS Study. J Bone Miner Res,. 2004;19(5):782-93.
14. McCloskey E, Selby P, Davies M, Robinson J, Francis RM, Adams J, et al. Clodronate reduces vertebral fracture risk in women with postmenopausal or secondary osteoporosis: results of a double-blind, placebo-controlled 3-year study. J Bone Miner Res,. 2004;19(5):728-36.
15. Simons LA, McCallum J, Simons J, Powell I, Ruys J, Heller R, et al. The Dubbo study: an Australian prospective community study of the health of elderly. Australian and New Zealand journal of medicine. 1990;20(6):783-9.
16. McInerney-Leo AM, Schmidts M, Cortes CR, Leo PJ, Gener B, Courtney AD, et al. Short-rib polydactyly and Jeune syndromes are caused by mutations in WDR60. Am J Hum Genet. 2013;93(3):515-23.
17. Li H, Homer N. A survey of sequence alignment algorithms for next-generation sequencing. Briefings in bioinformatics. 2010;11(5):473-83.
18. Li H, Handsaker B, Wysoker A, Fennell T, Ruan J, Homer N, et al. The Sequence Alignment/Map format and SAMtools. Bioinformatics. 2009;25(16):2078-9.
19. McKenna A, Hanna M, Banks E, Sivachenko A, Cibulskis K, Kernytsky A, et al. The Genome Analysis Toolkit: a MapReduce framework for analyzing next-generation DNA sequencing data. Genome Res. 2010;20(9):1297-303.
20. DePristo MA, Banks E, Poplin R, Garimella KV, Maguire JR, Hartl C, et al. A framework for variation discovery and genotyping using next-generation DNA sequencing data. Nat Genet. 2011;43(5):491-8.

21. Wang K, Li M, Hakonarson H. ANNOVAR: functional annotation of genetic variants from high-throughput sequencing data. Nucleic acids research. 2010;38(16):e164.
22. Abecasis GR, Altshuler D, Auton A, Brooks LD, Durbin RM, Gibbs RA, et al. A map of human genome variation from population-scale sequencing. Nature. 2010;467(7319):1061-73.
23. Albers CA, Lunter G, MacArthur DG, McVean G, Ouwehand WH, Durbin R. Dindel: accurate indel calls from short-read data. Genome Res. 2011;21(6):961-73.
24. Morris JA, Kemp JP, Youlten SE, Laurent L, Logan JG, Chai RC, et al. An atlas of genetic influences on osteoporosis in humans and mice. Nature Genetics. 2019;51:258-66.
25. de Leeuw CA, Mooij JM, Heskes T, Posthuma D. MAGMA: generalized gene-set analysis of GWAS data. PLoS computational biology. 2015;11(4):e1004219.
26. Pruim RJ, Welch RP, Sanna S, Teslovich TM, Chines PS, Gliedt TP, et al. LocusZoom: regional visualization of genome-wide association scan results. Bioinformatics. 2010;26(18):2336-7.
27. Xiao J, Purcell SA, Prado CM, Gonzalez MC. Fat mass to fat-free mass ratio reference values from NHANES III using bioelectrical impedance analysis. Clinical Nutrition. 2018;37(6, Part A):2284-7.
